# Reproductive and child health transition among selected empowered action groups states of India: A district-level analysis

**DOI:** 10.1371/journal.pone.0301587

**Published:** 2024-06-10

**Authors:** Bindhy Wasini Pandey, Ganesh Yadav, Niharika Tripathi, Praveen Kumar Pathak

**Affiliations:** 1 Department of Geography, Delhi School of Economics, University of Delhi, New Delhi, India; 2 Department of Geography, Kalindi College, University of Delhi, New Delhi, India; 3 Department of Sociology, Indraprastha College for Women, University of Delhi, New Delhi, India; 4 Centre for the Study of Regional Development, Jawaharlal Nehru University, New Delhi, India; State Health Resource Center, Chhattisgarh, INDIA

## Abstract

**Background:**

Health is an inseparable part of life and central to all life supporting systems. The reproductive and child health shares a major portion of public health cases that is crucial for socio-economic development. Studies on reproductive and child health have traditionally been focused on demographic aspects using socio-economic parameters. Given the emphasis of Sustainable Development Goal (SDG)-3 on health and well-being, it is imperative to understand the geo-spatial dimension with the visible transition of key health indicators of fertility, maternal and infant/child health in the high burdened districts within these high focus Empowered Action Group (EAG) states of Rajasthan, Madhya Pradesh, Uttar Pradesh and Bihar that make up nearly 40% of India’s population with relatively laggard health status.

**Methodology:**

This paper aims to understand the status and trend of key reproductive and child health indicators and vital statistics based on the recent representative demographic surveys. We intend to undertake a district level spatio-temporal analysis by developing District Composite Health Profile (DCHP) using Composite Index Method on selected 13 equally weighted key reproductive and child health indicators. The study has been carried out using data from National Family Health Survey-4 (2015–16) and National Family Health Survey-5 (2019–21) survey rounds. We employed geo-spatial techniques i.e. Moran’s–*I*, and univariate LISA to comprehend the geographical clustering of high and low health burden districts and their heterogeneities at the district level.

**Results/Conclusions:**

The study highlights emerging inter-districts, and inter-state disparities over survey periods. With consistent improvement in the selected EAG states over time, the overall reproductive and child health status through DCHP along with each indicator was relatively better in the states of Rajasthan and worse in Bihar. Districts along the Terai belt in Uttar Pradesh and Bihar consistently performed sluggish during survey rounds. The geo-spatial clustering follows the political boundary of states, albeit with intra-state variations. Monitoring of key health indicators using composite index method provides a useful leverage for identifying priority districts/regions for universal health access that should also consider geographical space as an important policy dimension.

## Introduction

Health is an intrinsic part of human life which is central to all the life supporting systems and survival. Good health and well-being of masses irrespective of race, colour, gender and nationality is the principal theme of Sustainable Development Goal (SDG 3) [1, 2]. Traditional studies have largely focussed on understanding the role of socio-economic constructs of maternal morbidity and mortality, infant and child survival and fertility preferences without much acknowledging the potential role of geographical space using index based approaches [3–8]. There are spatial differentials in terms of health indicators and their broad domains like fertility, mortality, reproduction and family planning, maternal health and care, and child health and care, etc. [9]. India scores rather unsatisfactory on the key indicators of maternal health, infant health, fertility behaviour and overall reproductive health with significant spatial variations at regional and sub-regional levels [9, 10]. These inequalities are defined as differences in terms of health status and factors that determine health as per populations or spatial units. This inequality is unfair or unjust from socio-economic point of view [2, 11, 12]. The different spatial patterns to this inequality adds another layer to it [12, 13]. Inequality leads to iniquity that further leads to disproportionate access to facilities [11]. These disparities in health outcome are major impediments to realising sustainable Development Goals, reducing mortality and morbidity, and achieving the universal health care [14–16]. The Empowered Action Group (EAG) states in India is a group of states with rather unsatisfactory performance of reproductive and child health indicators, low income or wealth levels, and overall tragic health outcomes. Health inequality burden is also comparatively high in these EAG states [17–20]. Over the years there has been a significant drop in the fertility in the major EAG states i.e. Bihar, Rajasthan, Madhya Pradesh and Uttar Pradesh as it has dropped significantly in Uttar Pradesh from 4.06 in NFHS-2 to 2.74 in NFHS-4; in Bihar from 3.70 (NFHS-2) to 3.41 (NFHS-4); in Rajasthan from 3.78 (NFHS-2) to 2.40 (NFHS-4); and in Madhya Pradesh from 3.43 (NFHS-2) to 2.31 (NFHS-4) [21]. This fertility transition is closely associated with socio-economic transformation in these states and their districts [22, 23]. The overall under nutrition incidence has decreased among EAG states over the last decade but wasting component has not improved for the same period [17]. Among many factors, maternal education, household wealth and place of residence were important confounders to inequality in undernutrition for children from 2006 to 2016 [9, 18, 19]. Studies show that substantially higher burden of under 5 mortality rates were observed in EAG states in comparison to other states, nevertheless, there has been improvements [9, 17–19, 23, 24]. Besides, there are visible rural-urban differentials in terms of Infant mortality rates improvement in these states of India [19]. But there are several determinants other than socio-economic confounders that play important role in determining child’s health [10, 25]. For example, the planning for child survival program in rural EAG states undertakes parental competence explaining the unobserved familial effect along with other program based determinants [15, 18].

On the key maternal health indicators i.e. Anti Natal Care (ANC), Post Natal Care (PNC), institutional births, births assisted by skilled persons, level of anaemia, etc., the performance of EAG states is tragically truncated in comparison to other states of India [20, 21, 26, 27]. Majority of studies on India have traditionally been focused on socio-economic and health related determinants of maternal health [28, 29]. The Maternal Mortality Ratio (MMR) in Bihar, Rajasthan, Madhya Pradesh and Uttar Pradesh were 165, 188, 186, and 216 per 1,00,000 live births for the period 2015–2017, worse than the national average [30, 31]. Similarly, in terms of nutritional deficiency i.e. status of anaemia and Weight-for-Age (underweight) parameter for women, the bigger EAG states i.e. Bihar, Uttar Pradesh, Rajasthan and Madhya Pradesh have performed poorer than even to rest of EAG states [5, 21, 26].

The reproductive and child health analysis using indicative approach is often convenient for socio-economic purposes but regional or spatial approach is a strong tool to comprehensively understand the effect of geographical space [32, 33]. The association between geographical attributes and demographic health can be helpful in health policy and management as aggregation of health variables over the space yield striking clustering, that if ignored can diminish the effectiveness of association. In recent years, a number of public health studies have focussed on understanding health in the geographical space due to increasing impact of contextual factors [10, 19, 34, 35]. The application of advanced Geographical Information System (GIS) techniques have made this possible to show the effect of spatial diffusion or differentiation on particular health parameters and their association with other geographical variables [24, 36–38]. This is perhaps the only study of its kind that uses composite index method for understanding outcomes and patterns over geographical space to assess the reproductive and child health and its transition in the EAG states. Literatures indicate that some studies used crude arithmetic as well as robust statistical methods to aggregate indicators for obtaining the health based composite scores [36, 39, 40]. Studies using composite index have been used to study and monitor the health coverage, health policy and planning, and nutritional status and many of them are much relevant in Indian context given wide geographical coverage and broad population base [7, 8, 36, 41–43].

## The study area

**Fig 1:**
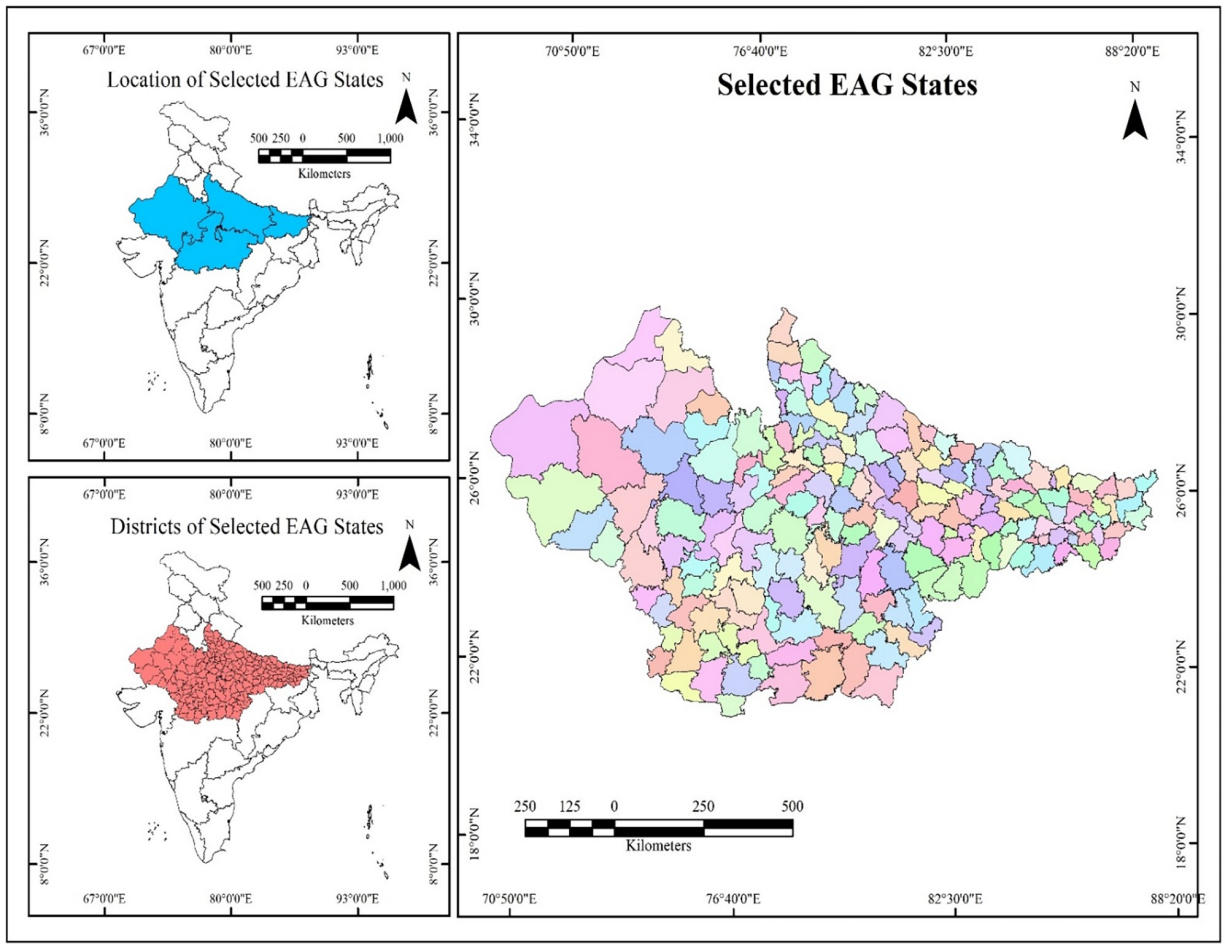
The study area. Source: Prepared by authors.

The EAG states are designated as high focus states with persistent low scores on various health and economic parameters over the years [44]. There are a total of eight EAG states namely Bihar, Chhattisgarh, Jharkhand, Odisha, Rajasthan, Madhya Pradesh, Uttarakhand and Uttar Pradesh. Our study is restricted to Bihar, Rajasthan, Madhya Pradesh, and Uttar Pradesh, which have been exhibiting conspicuously high fertility, high IMR, high MMR, high population growth rate, low literacy and low per capita income levels and GDP over the years [45–49]. The selected EAG states are the largest in terms population size, making up to 80 percent among EAG states and nearly 40 percent of India’s total population [45, 50]. Uttar Pradesh has the highest birth rates in India at 29.3 on the other hand Madhya Pradesh is reported to have the highest infant Mortality rate at 48, under five mortality rates at 56 and highest neo natal mortality at 35. Bihar has the highest total fertility rates and live births attended by untrained functionaries at the time of delivery in India [47]. Uttar Pradesh has also the second highest burden of maternal mortality rates in India [30]. Geographically, these four states represent four different regions as well i.e. Uttar Pradesh is largely an agrarian state; Rajasthan has desert like conditions; Madhya Pradesh is largely tribal and forested state whereas Bihar is a flood prone (**Fig 1**).

## Materials and methods

### Ethics statement

The study was based on an anonymous public use data set with no identifiable information on the survey participants, therefore no ethics statement is required.

### Data

This study is based on data from two successive rounds of nationally representative survey i.e. National Family Health Survey (NFHS)- 4 (2015–2016), and NFHS 55 (2019–2021). NFHS is an Indian version of Demographic and Health Surveys (DHS), that provides consistent and reliable information at district level corresponding to the household primarily on fertility, mortality, contraception uses, health care utilisation and other demographic health estimates [21, 26]. NFHS is based on a multi stage stratified clustering sampling design using four different type of questionnaires on general household information, eligible women health, eligible men health and biomarker like height and weight. In the first stage of sampling plan, population are stratified in to urban and rural clusters in a state, the sampling size of which is proportional to the respective state population. Primary sampling units are villages and towns in the rural and urban areas respectively and they were selected based on the probability proportional to the population size. This is followed by random selection of households within the primary sampling units. The selection of samples for questions related to women and child health is based on the presence of eligible women aged 15–49. For assessing the overall reproductive and child health, we have developed a composite index i.e. District Composite Health Profile (DCHP), calculated on 13 indicators related to fertility, women and infant health at the district level on both NFHS-4, NFHS-5 rounds data. Appropriate weights were assigned before obtaining results to make the estimates representatives and comparable over the regions of analysis. The details of weights are provided in the NFHS reports of all the rounds. Analysis of vital statistics i.e. birth rates, death rates, MMR and IMR have been done on the information taken from Sample Registration System (SRS) from 1997 to 2019. While understanding the transition of DCHP in the selected EAG states from2015-16 to 2019–21, the number of districts increased from 192 in NFHS-4 to 197 in NFHS-5. The data for these newly formed districts were imputed for NFHS-4 based on NFHS-5.

### Analytical strategy

DCHP composite index was developed on key reproductive and child health indicators relating to broader domains of fertility, maternal and child health. Higher DCHP is indicative of better reproductive and child health and vice versa. The methodology for calculating DCHP involves the derivation of composite index [36, 40, 51, 52], based on the equally weighted indicators because we intended to develop an assessment tool applicable to a diverse set of areas in diverse set of settings. This method is used by UNDP for calculating Human Development Index [40]. This approach is also used by NITI Aayog for assessing multidimensional poverty index at district level in the direction to implement robust SDG framework [53]. Table 1 represents the list of indicators, their domains and relationship with DCHP based on their nature. Percentage of currently married women (15–49) with total unmet need of Family Planning indicate the percentage of currently married women willing to delay their next birth for 2 or more years or stop childbearing altogether, but are not using a contraceptive method, or have a mistimed or unwanted current pregnancy or their last birth in the last two years is unwanted. A higher proportion indicates higher need for using contraceptives that remains unfulfilled suggesting high fertility behaviour, risky pregnancies and undermined health of mother and child [54]. Similarly, percent of currently married women (15–49) using any method of Family Planning suggests the proportion of women who have access to contraceptives as access and usage of these methods help in explaining the regulation of fertility behaviour, and safe pregnancies [22]. The percentage of women who had four or more ANC visits has been taken as an important indicator from maternal health domain, as ante-natal care visits have been found to be highly associated with reduced maternal mortality, neo natal mortality, and pregnancy failures [55, 56].

**Table 1:** Indicators, domains and their relationship with District Composite Health Profile (DCHP).

Domains	Indicators	Relationship with DCHP
Fertility	Percentage of currently married women (15–49) with total unmet need of Family Planning	(-) Ve
	Percent of currently married women (15–49) using any method of Family Planning	(+) Ve
Maternal Health	Percentage of Women who had four or more ANC visits	(+) Ve
	Percentage of women having any anaemia (<12.0 g/dl)	(-) Ve
	Percentage of Women with BMI <18.5 (total thin)	(-) Ve
	Percentage of Births delivered in a health facility	(+) Ve
	Percentage of Deliveries assisted by health personnel	(+) Ve
	Percentage of Births with three and more children	(-) Ve
Child Health	Percentage of children with full vaccination	(+) Ve
	Percentage of Children with Height for Age (Stunting) below -2 SD	(-) Ve
	Percentage of Children with Weight for Height (Wasting) below -2 SD	(-) Ve
	Weight for Age for Weight (Underweight) below -2 SD	(-) Ve
	Percentage of Children having any anaemia (<11.0 g/d)	(-) Ve

Source: Prepared by authors, 2022.

The percentage of women with BMI <18.5 (total thin) and percentage of women having any anaemia (<12.0 g/dl) are suggestive to the undernutrition and studies highlight that these are closely associated with child undernutrition and maternal mortality [57, 58]. Apart from physiological factors, health facilities and attendance by skilled health professional play critical role in minimising the mortality and morbidity burden [25, 59, 60]. Percentage of births delivered in a health facility and percentage of deliveries assisted by health personnel have been taken as indicators to incorporate health facility in to the composite score. Number of children pf a mother have a major implication on maternal pregnancies and post-natal characteristics and this association have been found to have long term maternal health consequences [61]. These indicators are also in consonance with SDG goal target 3.1 and 3.7 to reduce maternal mortality below 70 per 100,000 births and universal access to sexual and reproductive health-care services, including for family planning, information and education [2].

From child health domain, percentage of children with full vaccination is an important lifesaving component to reduce the child mortality and morbidity [62]. The SDG-3, target 3.2 aims at ending preventable deaths of newborns and children under 5 years of age by 2030. Besides, target 3.8 aims to achieve universal health coverage, including access to quality essential health-care services and access to safe, effective and affordable essential medicines and vaccines for all [2]. All the three components of child nutrition i.e. stunting, wasting and underweight along with percentage of children having any anaemia (<11.0 g/d) have been used as indicators of child health as this has been revealed in numerous studies that nutrition plays an important role in morbidity free years of human life [58, 63, 64]. There might be other indicators to justify the reproductive and child health but we have taken only those indicators those data are readily available and reliable at global standards. All these indicators were calculated in to percentage form to show prevalence using standard nominators and denominators for the reference population for all the districts using DHS manual on STATA 16 statistical package [26].

For calculating the DCHP, data of each indicator were prepared as per their relation with DCHP. The data preparation involved calculating the percentage prevalence of each indicator for each district from large size samples. This was done using the relevant nominators and denominators. The appropriate weights were assigned before calculating the prevalence. Once the district wise prevalence each indicator was obtained for all 197 districts, the data set was reorganised as per their relationship with DCHP. Indicators having positive relationship with DCHP were taken as it was but the indicators with negative relationship with DCHP were reversed as per its corresponding value at that position for the referenced district. For example, a greater percentage of Full vaccination (Positive to DCHP) in a district would result in higher DCHP implying better condition of reproductive and child health condition, on the contrary, a higher percentage of wasting among children (Negative to DCHP) will effectively erode DCHP, therefore, such negative indicators were reversed. This was followed by standardization of Indicators to solve the unit measurement issues. This step was aimed at neutralizing the scale effect to make them standard and comparable with each other [52]. This was calculated using the following formula;

$$IND^{v}=\frac{I-Min^{*}(I)}{Max(I)-Min^{*}(I)}$$

Where,

 IND^v^ is the standardized score of each indicator

 I is the indicator value for v^th^ district.

 Min*(I) minimum value of indicator.

 Max (I) maximum value of indicator.

IND^v^ is standardized value and satisfies 0 < *IND* ^*v*^ ≤1. The min*(*I*) and max (*I*) have been referred to as “goalposts”. This minimum and maximum values of indicator is the lowest and highest value district among whole values for all 197 districts under the study. Min*(I) should strictly be the minimum among the observed distribution which make the IND^v^ > 0 and choosing Min*(I) is defensible in decision making. This also holds true in case of maximum value respectively [40, 51]. The final DCHP values for each district was obtained by amalgamation of Standardized Indicators by taking the geometric mean for each district v.


$${\text{DCHP}}^{v}=\sqrt[{n}]{\overset{n}{\underset{i=1}{\prod }}INDv}$$

Where,

DCHP^v^ District Composite Health Profile of v district

Π Geometric mean

INDv All Indicator scores of v district.

n Number of Indicators in observation.

The advantage for choosing geometric mean over other means is that a percentage change in any indicator has the same effect on the geometric mean, regardless of the indicator’s range. Another important advantage of using this method is that all indicators are given equal weightage, particularly the smaller values. The DCHP values for a unit thus reflects that unit’s (geometric) average proportional distance from the lower goalpost, min*(*I*), which is its most important property [40].

The spatial analysis of DCHP along with its indicators was done at the district level. We adopted the district boundaries corresponding to two different rounds of National Family Health Survey data downloaded from the Demographic Health Survey (DHS) spatial data repository based on 2011 Indian Census. The new districts that emerged between NFHS-4 to NFHS-5 were carved from their parent district/s using the same 2011 Indian Census publication. The basic rationale behind using district boundary was to highlight the inter-district disparities and similarities within these high focus states. Further, district unit is the smallest administrative entity where all the development and social benefit programs are implemented by the Government of India and state’s various government agencies. Therefore, monitoring of population health parameters using composite indices at the district level provides key leverage on policy and programs design, monitoring and implementation. Another rationale for using district level analysis is to demonstrate the role of geographic space as an independent factor contributing towards demographic health based on maternal, child and family planning variables. The sample sizes of these districts are sufficient enough to provide strong estimates on various variables under the study [65]. All 197 districts were chosen for generating composite maps and spatial analysis.

**Fig 2:**
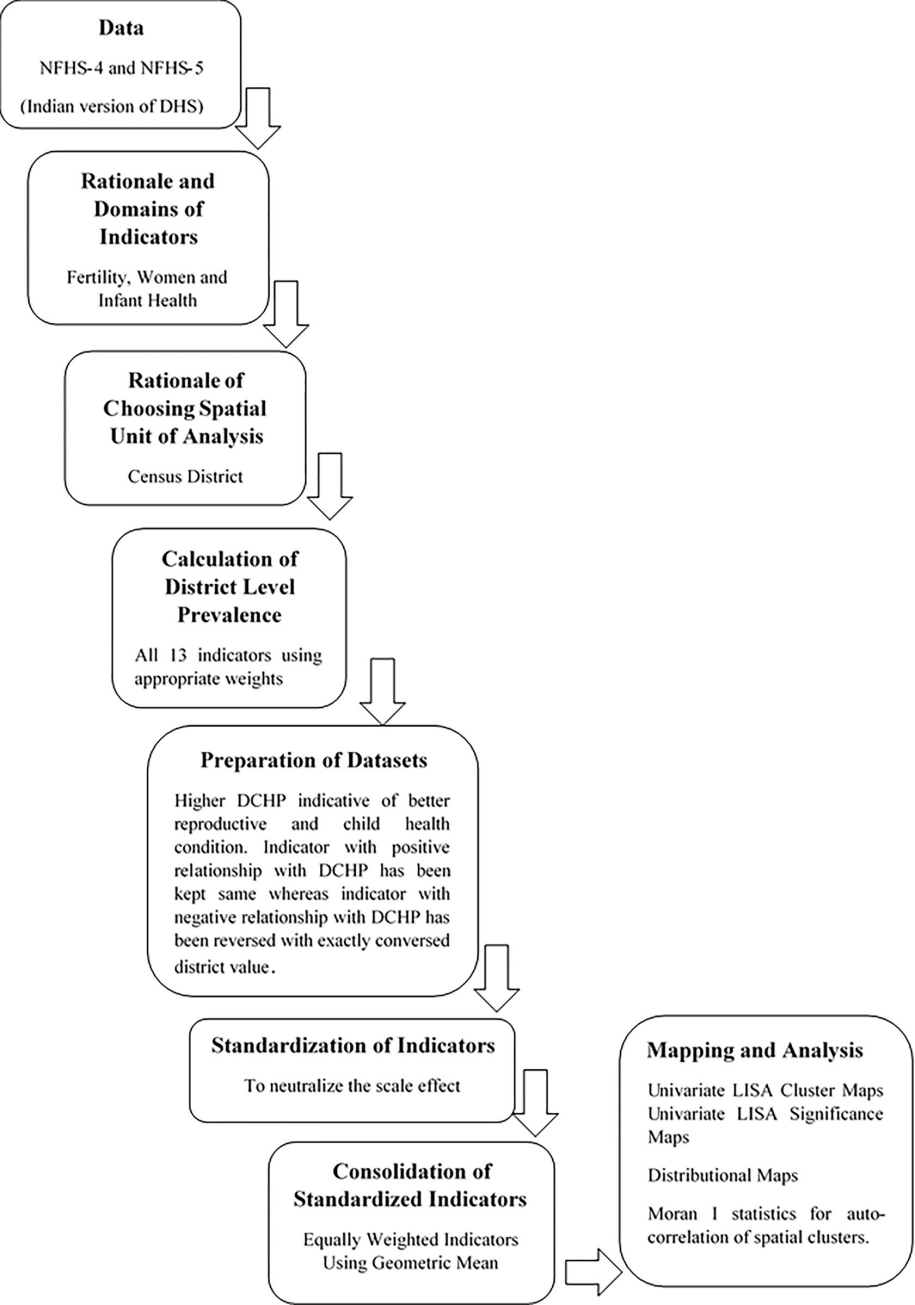


We used ArcGIS package to generate DCHP4 (2015–16) and DCHP5 (2019–21) choropleth maps across all the study districts. The shape files were then exported from ArcGIS to GeoDa software environment for advanced spatial analysis. GeoDa is the software tool suitable for various exploratory spatial data analysis including data manipulation, mapping and other advanced geo-statistical applications [66, 67]. Spatial weights were generated using GeoDa, essentially required for the computation of spatial autocorrelations. We chose contiguity based spatial weights since our main aim was to understand spatial interdependence of indicators. Rook’s contiguity weights were used for estimating all the geo-spatial statistics and auto-correlations that uses common boundaries to define neighbours. The relevant geo-spatial techniques like Univariate LISA and Moran-*I* statistics was used to explore the research questions. The Univariate LISA automatically makes clusters for one indicator. The LISA cluster map yields four major types of clustering i.e. high-high, high-low, low-high and low-low. ‘High-high’ category means districts with above average scores share boundaries with neighbouring districts that have above average scores of the variables of interest. On the contrary, ‘high-low’ means that districts with above average scores share the boundaries with districts with below average scores. Similarly, ‘low-high’ districts have below average scores and these are surrounded by the districts with above average scores. The low-low’ category districts are characterised by below the average scores that share the boundaries with the districts with below average scores. The ‘high-high’ are referred to as *hot spots*, whereas the ‘low-low’ are referred to as *cold spots* [10, 68, 69]. The Moran’s *I* is used to see the degree of auto-correlation of such spatial clusters. Moran’s *I* is a measure of spatial autocorrelation using Pearson coefficient method [70]. It measures the degree to which data point are similar or dissimilar to their spatial neighbours. Positive autocorrelation indicates how points with similar attribute values are closely distributed, whereas negative spatial autocorrelation are means how closely associated points are not similar. Moran’s I value range from -1 (perfect dispersion) to +1 (perfect correlation), whereas 0 value indicates a random spatial pattern [71]. This was calculated using the following formula;

$$I\;=\;C\times \frac{\sum_{ij}W_{ij}Z_{i}Z_{j}}{\sum_{i}Z_{i}^{2}}$$

where Z_i_ is the standardized variable of interest; W_ij_ is the weight matrix; C is the ratio of total spatial units and the sum of all spatial weights, with *i* and *j* are the units or features of analysis. Detailed description of the methodological approach used in the study is presented in **Fig 2**.

## Results

### Progress of population health parameters in India and selected EAG states

**Fig 3:**
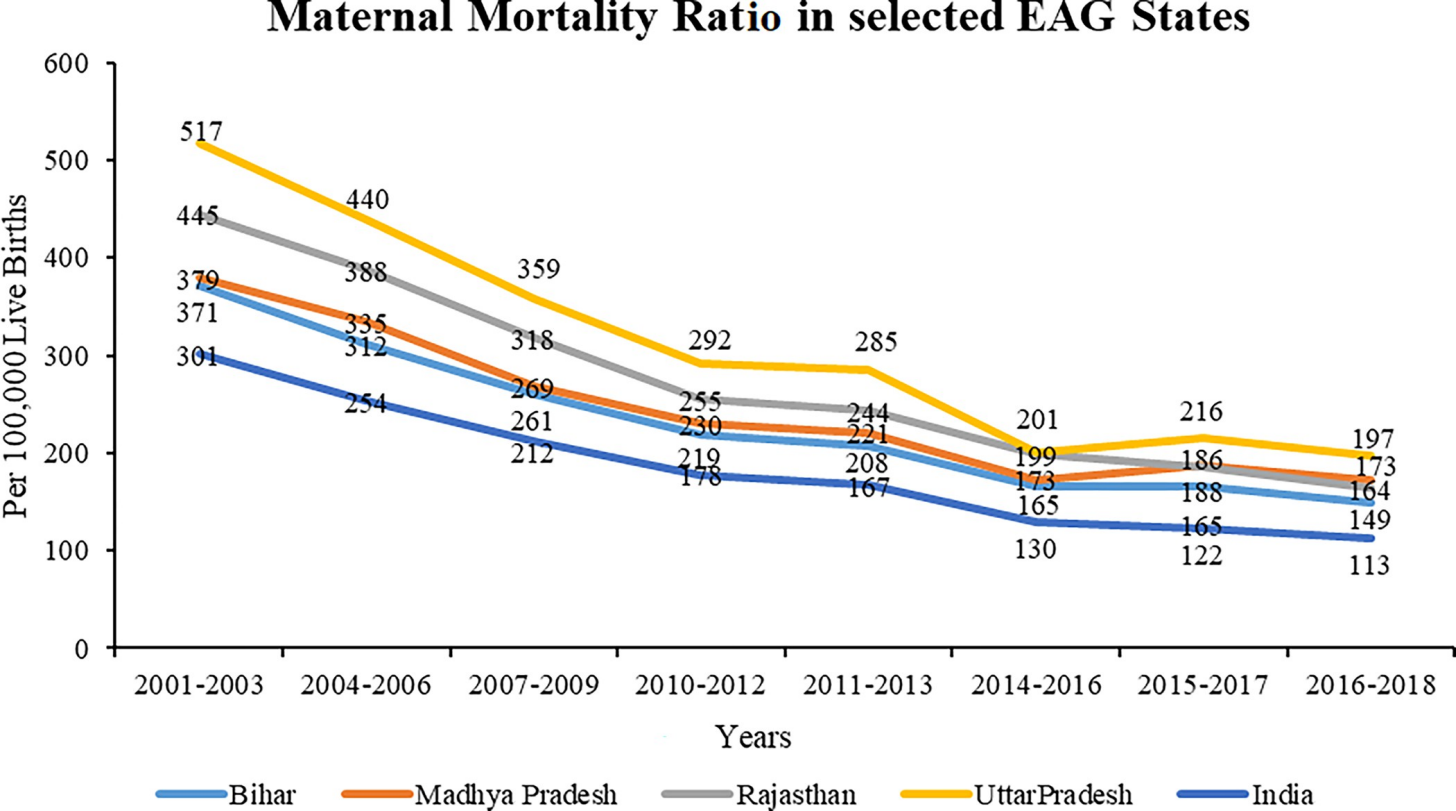
Maternal Mortality Ratio (MMR) in the selected EAG states (2001–2018). Source: Sample Registration System Bulletins, Office of Registrar General of India (2020). *Analysis for the year 2016–18 excludes data of Jharkhand, Chhattisgarh, and Uttarakhand in their parent states.

Trends of MMR suggests that all the selected EAG states have recorded consistently higher burden of maternal mortality, more than the national average between 2001–03 to2016-18 [27]. Uttar Pradesh has persistently recorded higher MMR among EAG states, followed by Rajasthan till 2015–17 when Madhya Pradesh recorded more MMR thereafter. State of Bihar has the least MMR among the selected EAG states. Trend analysis shows that almost all EAG states under the study exhibit continuous decline until 2010, after which there is inconsistency in decline as MMR rises for the reference years 2011–13, and 2015–17. Most importantly, the national MMR has fallen consistently since 2001–03 (**Fig 3**). Only the state of Assam has the MMR higher than Uttar Pradesh at 229 deaths per 100,000 live births, the highest among Indian states [72].

**Fig 4:**
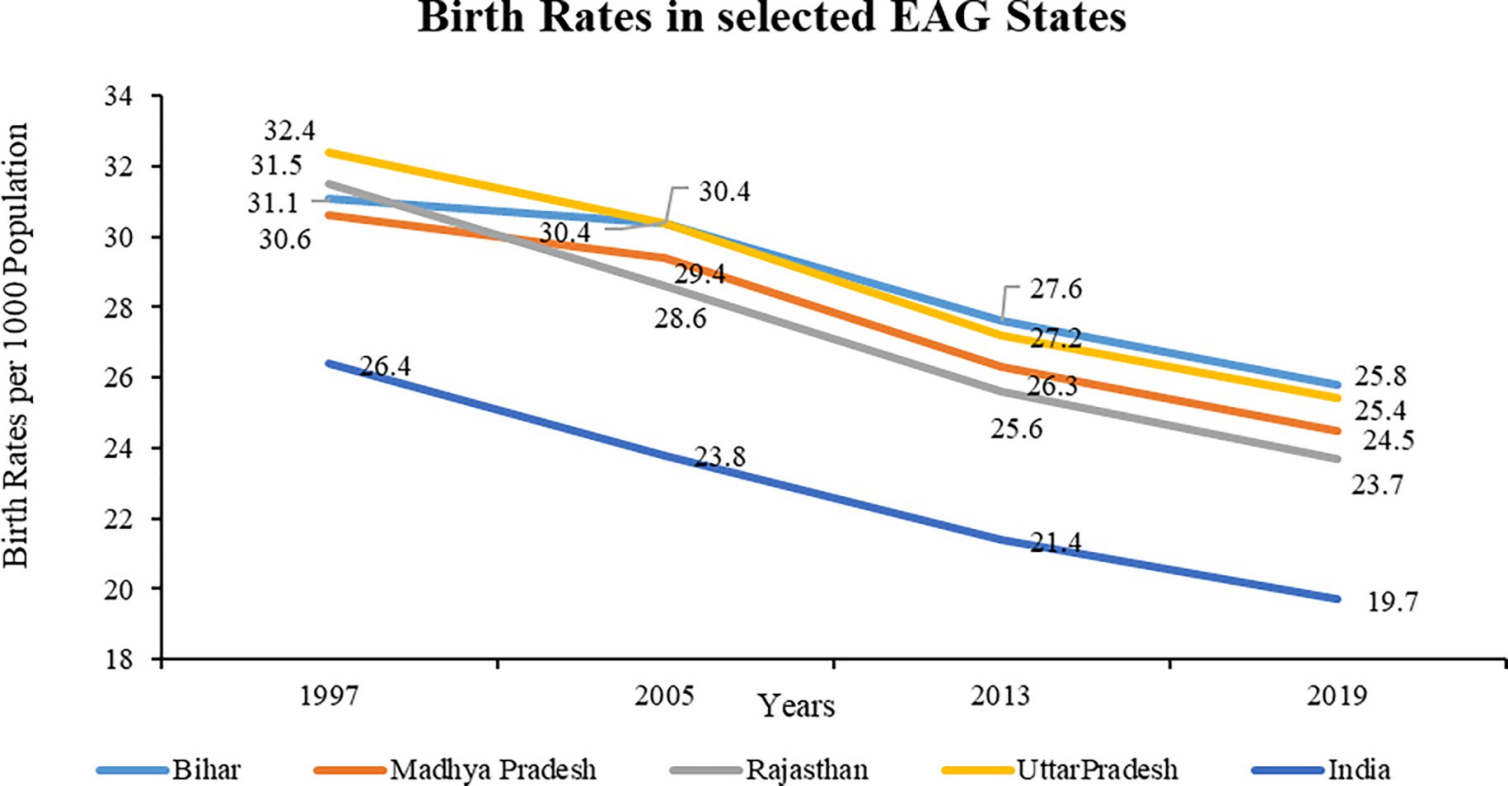
Birth Rates in the selected EAG states (1997–2019). Source: Sample Registration System Bulletins, Office of Registrar General of India (1999, 2006, 2014, 2021). *Analysis for the year 1997 includes data of Jharkhand, Chhattisgarh, and Uttarakhand for their parent states.

Birth rates (BR) are indicative of high fertility [71]. BR in the selected EAG states has been way above than the national average over the years. Uttar Pradesh has the highest BR in 1997. After 2005, Bihar has the highest BR, nonetheless, this has been falling afterwards. Rajasthan has consistently shown decline in BR since 1997 and has the lowest BR among selected EAG states at 23.7 in 2019. The decline in BR has been slow since 2013 in comparison to the reference period 2005–2013 (**Fig 4**).

**Fig 5:**
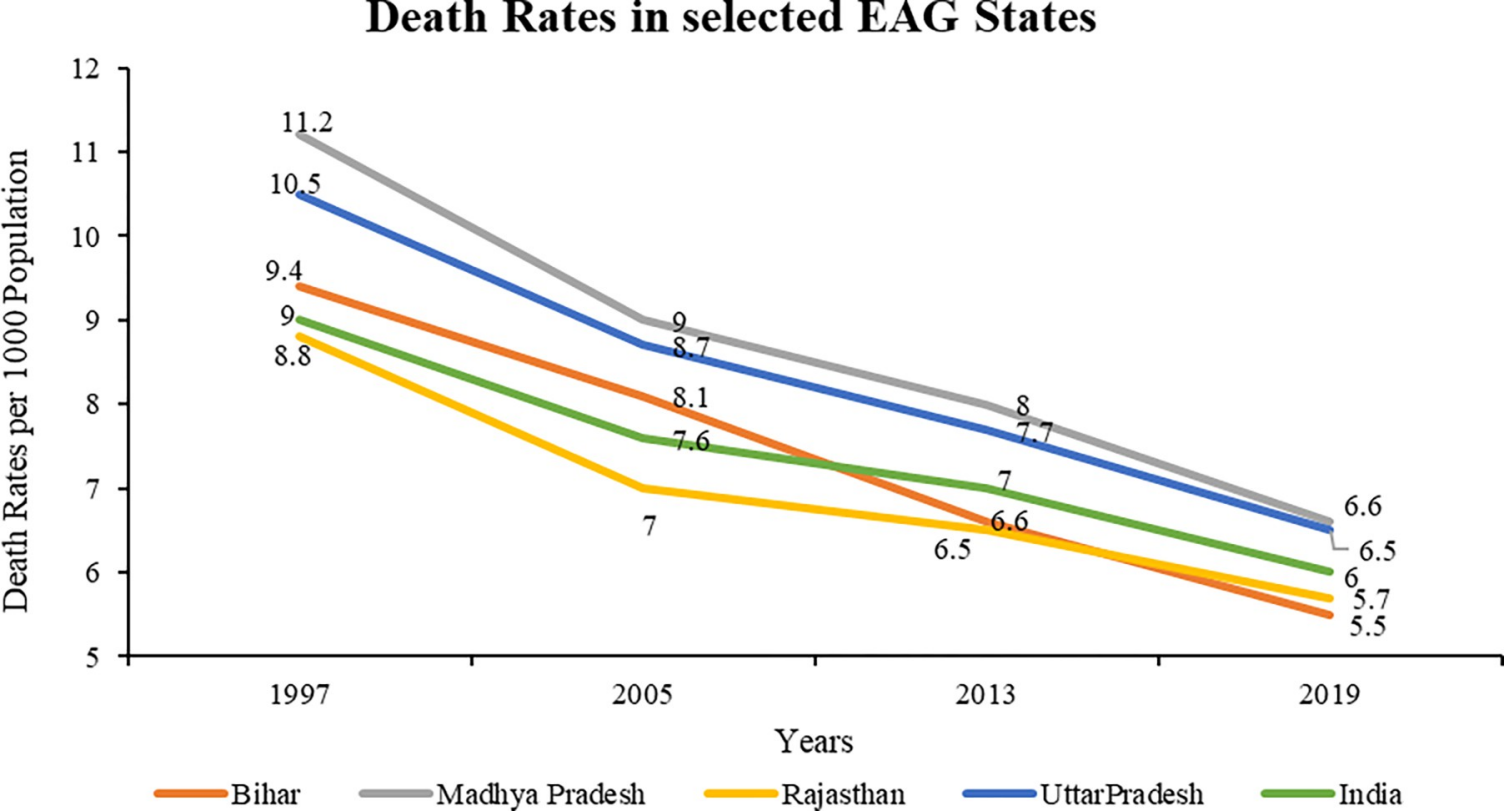
Death Rates in the selected EAG states (1997–2019). Source: Sample Registration System Bulletins, Office of Registrar General of India (1999, 2006, 2014, 2021). *Analysis for the year 1997 includes data of Jharkhand, Chhattisgarh, and Uttarakhand for their parent states.

Trends of Death Rates (DR) shows that Madhya Pradesh has the highest DR among the selected EAG states since 1997 till 2019 (**Fig 5**). The inter-state differences in DR among the selected EAG is higher in 1997 than in 2019, which shows that DR is averaging which can be explained by several similar socio-economic factors. DR has improved over the years due to various positive health interventions and improvements in health indicators [73].

**Fig 6:**
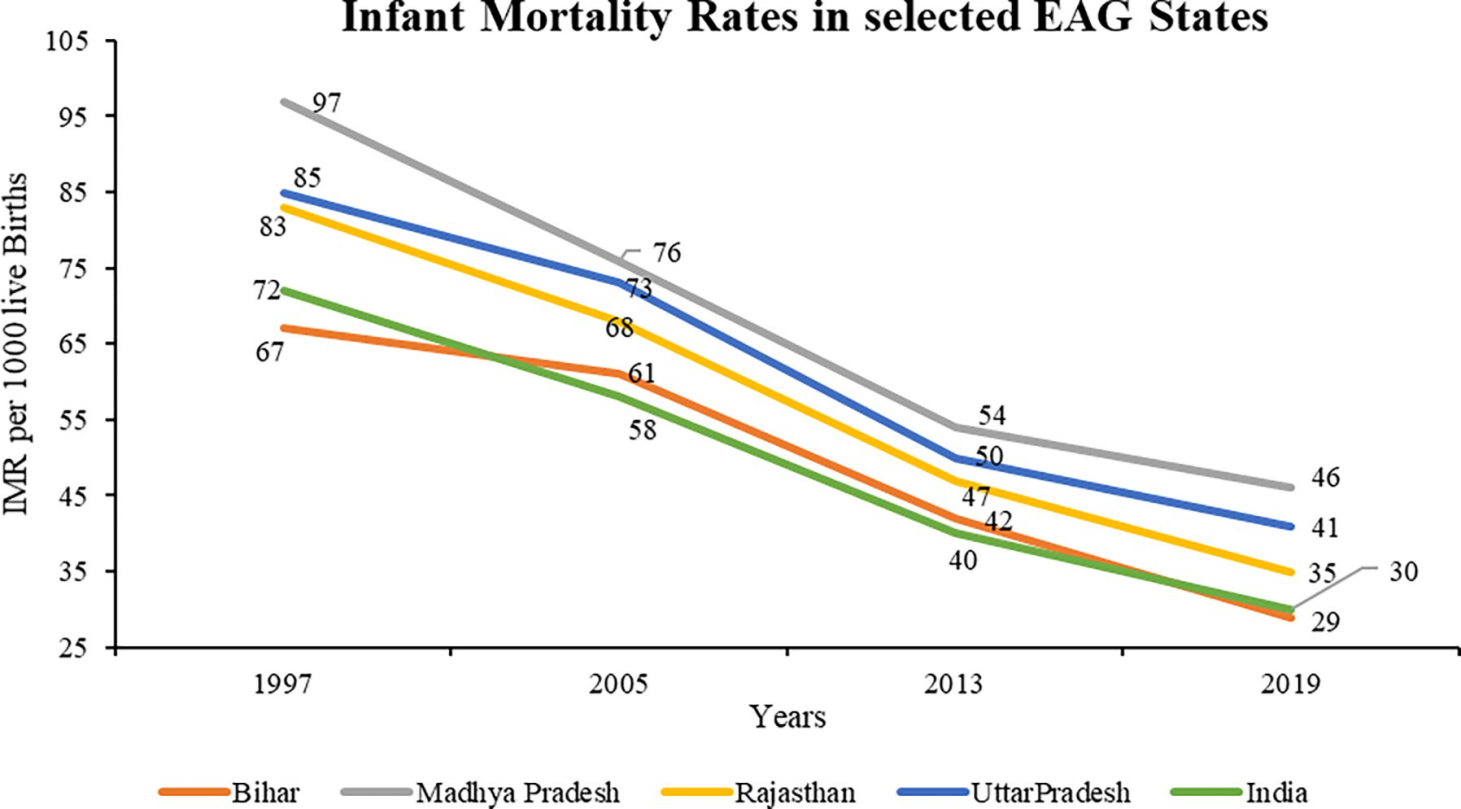
Infant Mortality Rates (IMR) in the selected EAG states (1997–2019). Source: Sample Registration System Bulletins, Office of Registrar General of India (1999, 2006, 2014, 2021). *Analysis for the year 1997 includes data of Jharkhand, Chhattisgarh, and Uttarakhand for their parent states.

Infant Mortality Rates (IMR) trends over the years indicate that selected EAG states have higher IMR than the national average. Madhya Pradesh has the highest IMR among the selected EAG states since 1997, followed by Uttar Pradesh, Rajasthan, and Bihar. All the selected EAG states have shown a steep decline since 1997 till 2013 after which the rate of decline has reduced. The reduction for the time period 1997 to 2005 in case of Bihar remained very slow (**Fig 6**). Despite significant improvement, IMR in India is alarmingly higher particularly among EAG states due to poor maternal health, lower education and wealth status [10].

### Analysis of district level population health parameters using District Composite Health Profile (DCHP)

**Fig 7:**
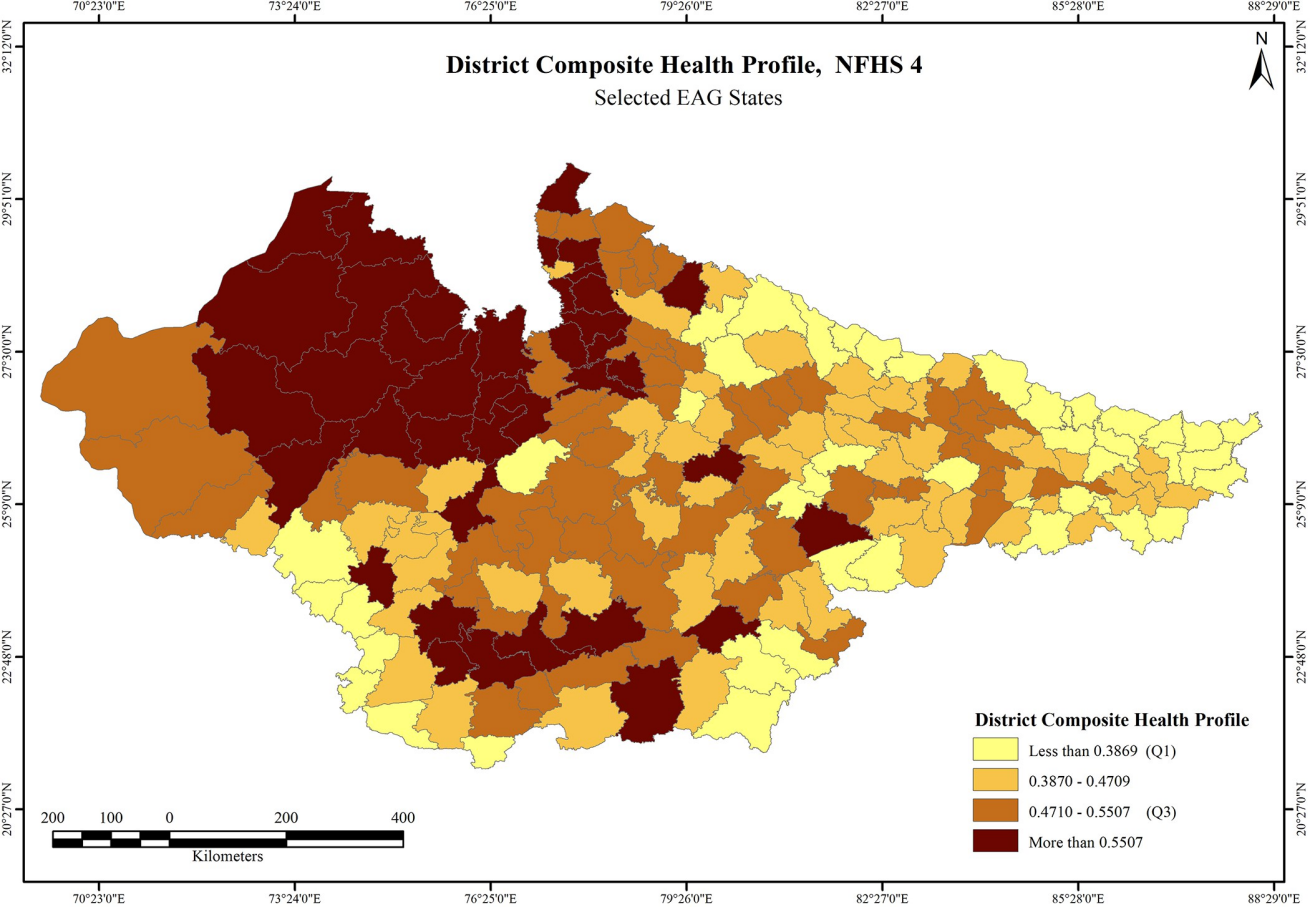
District Composite Health Profile, NFHS-4. Source: Prepared by authors based on NFHS-4, International Institute for Population Sciences (IIPS) and ICF. National Family Health Survey (NFHS-4), 2015-16, (2017).

**Fig 8:**
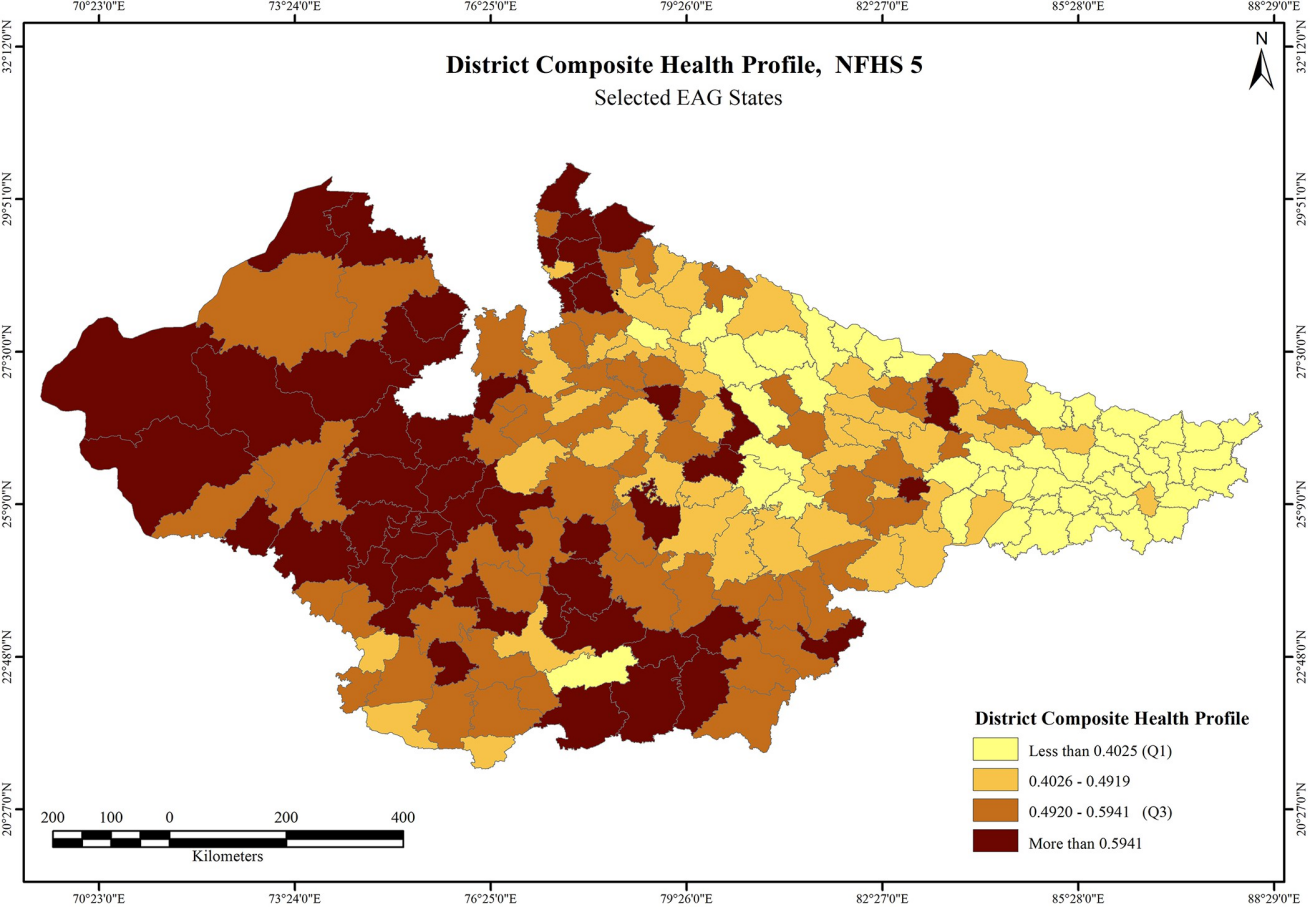
District Composite Health Profile, NFHS-5. Source: Prepared by authors based on NFHS-5, International Institute for Population Sciences (IIPS) and ICF. National Family Health Survey (NFHS-5), 2019-21 (2021).

The descriptive statistics for DCHP and its indicators is presented in the Table 2. The results of the final index values of DCHP across 197 districts were divided into four categories based on the quartile divisions. Results based on NFHS 4 indicates that most districts in central and eastern Rajasthan, western Uttar Pradesh and south Madhya Pradesh adjoining Indore, Bhopal, and Jabalpur fall within the more than Q3 category. Sri Ganganagar (0.8042) has the highest DCHP score, followed by Jhunjhunu (0.7822), and Jaipur (0.7598). In Madhya Pradesh, Indore (0.7283) has the highest score while Gautam Buddha Nagar (0.7246) has the highest score in Uttar Pradesh and Buxar (0.5260) in Bihar. Maximum number of districts in Bihar and Uttar Pradesh fall within the less than Q1 and median to Q1 category, particularly in the Terai region. Bahraich (0.1419), Shrawasti (0.1675), and Balrampur (0.1680) in Uttar Pradesh have the lowest scores (**S1 Table in S1 File**). No district in Bihar Falls within the category more than Q3, whereas maximum number of districts in Rajasthan, western Uttar Pradesh and central region of Madhya Pradesh have the highest DCHP scores (above Q3) (**Fig 7**). Findings on DCHP based on NFHS-5 indicates that higher number of districts in the above Q3 category has increased significantly, mostly in Rajasthan and Madhya Pradesh. Jaipur (0.7560) reported to have highest score, followed by Kota (0.7559), and Indore (0.7319). Bijnor (0.7157) has the highest score in Uttar Pradesh, whereas Siwan (0.4736) has maximum score in Bihar. Again, no district in Bihar falls within the more than Q3 category. The least scores in Bihar were reported from the districts of Purnia (0.1633), Katihar (0.1980), Araria (0.1991), and Saharsa (0.2129). Baharaich (0.2451) in Uttar Pradesh has the lowest score, followed by Balrampur (0.2548) whereas Hoshangabad (0.3929) and Daulpur (0.4745) have the lowest score in Madhya Pradesh and in Rajasthan, respectively (**S2 Table in S1 File**). The overall condition of reproductive and child health in the eastern Uttar Pradesh, most districts in Madhya Pradesh and central region of Rajasthan has significantly improved over 2016–21 period. It is important to note that a majority of districts falling in the Terai belt of Uttar Pradesh and Bihar have consistently performed poor with alarmingly low scores over the survey periods (**Fig 8**).

**Table 2:** Descriptive statistics of DCHP and its indicators based on NFHS-4 and NFHS-5.

	NFHS-4				NFHS 5			
Variables	Mean	Std. Dev.	Min	Max	Mean	Std. Dev.	Min	Max
District Composite Health Profile (DCHP)	0.4726	0.1208	0.1418	0.8042	0.4937	0.1304	0.1633	0.756
Percentage of currently married women (15–49)with total unmet need of Family Planning	45.32	17.009	2.7	74.6	65.46	11.48	25.3	83.4
Percentage of women who had four or more ANC visits	27.76	13.908	4.3	76.09	45.46	15.55	11.1	81.3
Percentage of Deliveries assisted by health personnel	73.97	13.355	30.73	97	86.44	8.93	54.6	99.3
Percentage of Births delivered in a health facility	75.67	11.937	36.7	98	87.15	7.69	64.9	99
Percentage of Births with three and more children	36.84	9.123	14.2	58.1	32.76	8.613	13.2	50.9
Percentage of children with full vaccination	54.9	12.837	7.1	79.9	74.5	9.99	44.5	93.7
Stunting	44.29	6.735	28.4	65.1	37.77	7.78	18	54.2
Wasting	21.9	6.655	8.4	39	19.41	5.62	8.6	36.8
Underweight	41.12	7.033	19.5	55	33.69	7.13	18.3	52.9
Percentage of children having any anaemia (<11.0 g/d)	64.61	10.095	38.7	84.8	69.44	8.47	37.8	87.2
Percentage of women having any anaemia (<12.0 g/dl)	53.77	10.144	25.9	76.3	55.24	8.4	33.8	75.2
Women with BMI <18.5 (total thin)	28.02	5.293	14.1	43.9	21.71	4.89	9.4	32
Percent of currently married women (15–49) with total unmet need of Family Planning	16.06	5.95	6.4	32.6	10.6	5.46	2.9	27.6

Source: Calculated by authors based on NFHS-4 and NFHS-5, International Institute for Population Sciences (IIPS) and ICF, 2015-16, 2019–21.

### Univariate LISA results

#### Outcome variables

**Fig 9:**
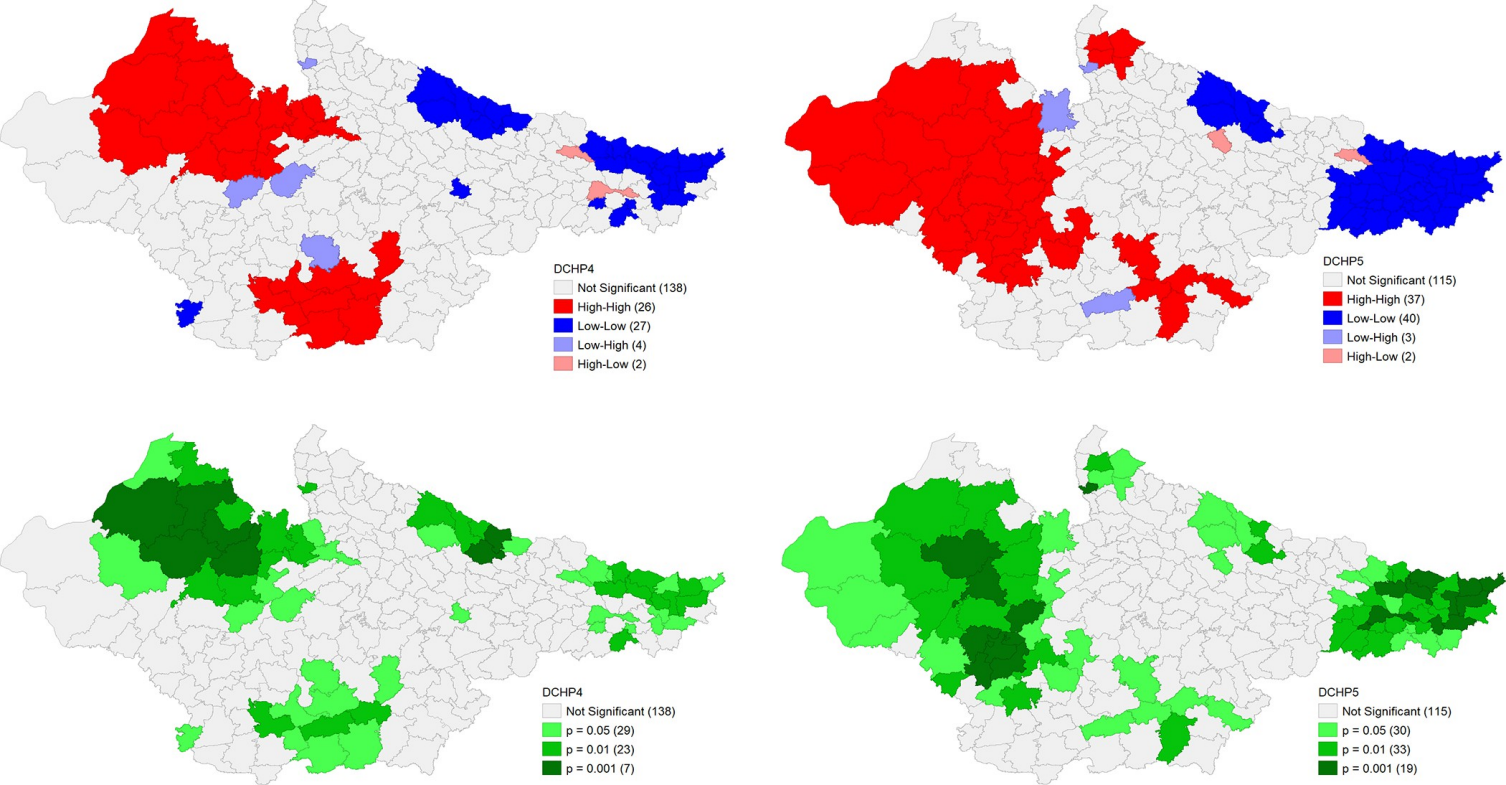
Univariate LISA (Cluster and significance) maps depicting spatial clustering and spatial outliers of District Composite Health Profile, based on NFHS-4 (DCHP4) and District Composite Health Profile, based on NFHS-5 (DCHP5). A. Univariate LISA Cluster map of DCHP4 across 197 districts in selected EAG States, 2015–16. B. Univariate LISA Cluster map of DCHP5 across 197 districts in selected EAG States, 2019–21. C. Univariate LISA Significance map of DCHP4 across 197 districts in selected EAG States, 2015–16. D. Univariate LISA Significance map of DCHP5 across 197 districts in selected EAG States, 2019–21.

The univariate LISA results for District Composite Health Profile based on NFHS-4 (DCHP 4) and District Composite Health Profile based on NFHS-5 (DCHP 5) are presented in Fig 8. There is substantial geographical clustering of high DCHP 4 districts in Rajasthan and central Madhya Pradesh districts. On the other hand, there is strong clustering of low DCHP 4 districts in northern and eastern Bihar and Terai belt districts of Uttar Pradesh. Clustering of these districts has become much robust in the NFHS 5 when almost all the districts of Rajasthan have high DCHP but slightly less in Madhya Pradesh. On the other hand, a strong clustering of low DCHP 5 districts is clearly visible in almost all the districts of Bihar but marginally reduced in case of Terai belt districts of Uttar Pradesh. The clustering of low DCHP 5 is statistically more significant than DCHP 4 in the districts of Bihar. Most districts of Bihar and Terai districts of Uttar Pradesh were characterised by truncated status of fertility, child health and maternal health indicators, in contrast to majority number of districts in Rajasthan and some districts of Madhya Pradesh around capital city Bhopal. This trend remained largely same in both the rounds (**Fig 9**).

### Indicators of DCHP

**Fig 10:**
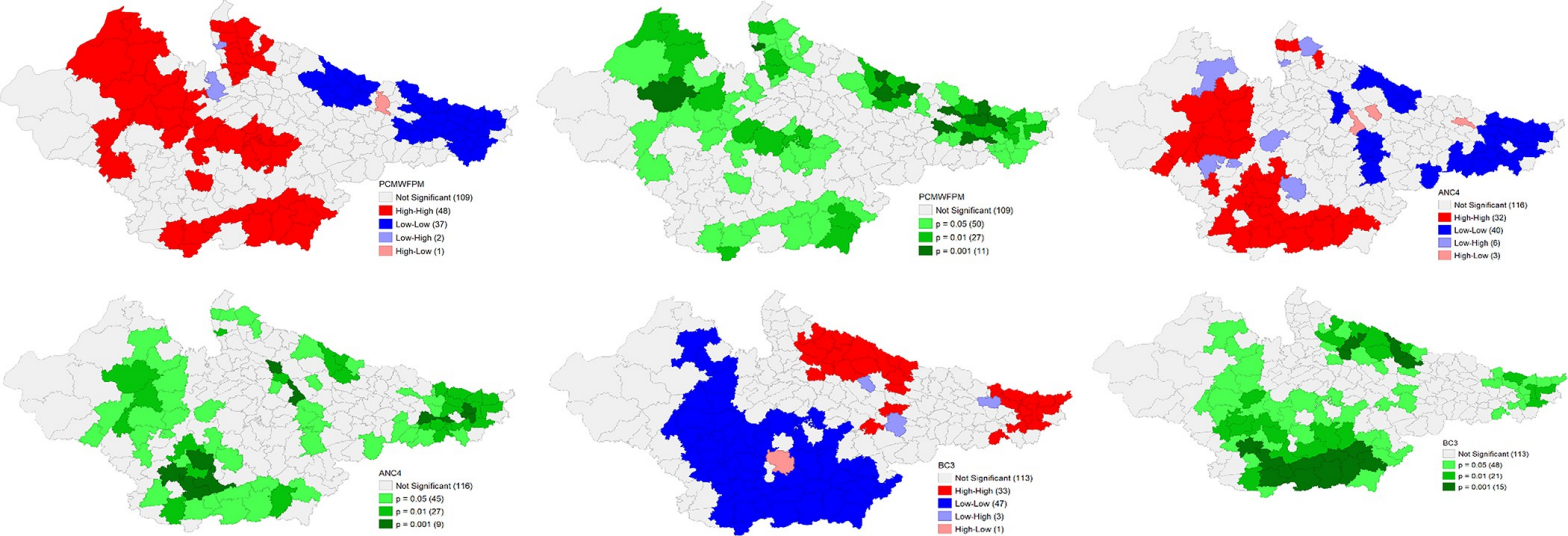
Univariate LISA (Cluster and significance) maps depicting spatial clustering and spatial outliers of District Composite Health Profile based on NFHS-4. A. Univariate LISA Cluster map of Percent of currently married women (15–49) using any method of Family Planning across 197 districts in selected EAG States, 2015–16. B. Univariate LISA Significance map of Percent of currently married women (15–49) using any method of Family Planning across 197 districts in selected EAG States, 2015–16. C. Univariate LISA Cluster map of Percentage of women who had four or more ANC visits across 197 districts in selected EAG States, 2015–16. D. Univariate LISA Significance map of Percentage of women who had four or more ANC visits across 197 districts in selected EAG States, 2015–16. E. Univariate LISA Cluster map of Percentage of Births with three and more children across 197 districts in selected EAG States, 2015–16. F. Univariate LISA Significance map of Percentage of Births with three and more children across 197 districts in selected EAG States, 2015–16.

**Fig 11:**
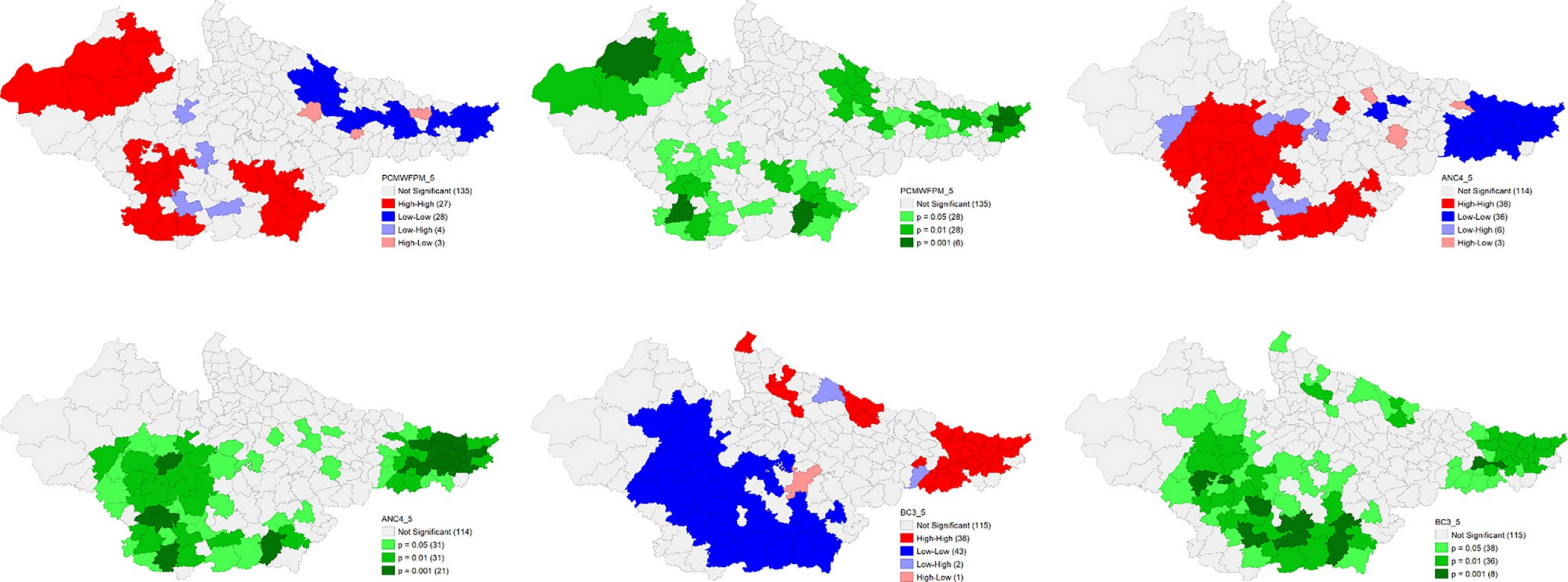
Univariate LISA (Cluster and significance) maps depicting spatial clustering and spatial outliers of District Composite Health Profile (DCHP 5), based on NFHS-5. A. Univariate LISA Cluster map of Percent of currently married women (15–49) using any method of Family Planning across 197 districts in selected EAG States, 2019–21. B. Univariate LISA Significance map of Percent of currently married women (15–49) using any method of Family Planning across 197 districts in selected EAG States, 2019–21. C. Univariate LISA Cluster map of Percentage who had four or more ANC visits across 197 districts in selected EAG States, 2019–21. D. Univariate LISA Significance map of Percentage who had four or more ANC visits across 197 districts in selected EAG States, 2019–21. E. Univariate LISA Cluster map of Percentage of Births with three and more children across 197 districts in selected EAG States, 2019–21. F. Univariate LISA Significance map of Percentage of Births with three and more children across 197 districts in selected EAG States, 2019–21.

The univariate LISA results for individual indicators of DCHP based on NFHS 4 and NFHS 5 are presented in the following figures that provide details of spatial clustering. Univariate LISA Cluster map of Percent of currently married women (15–49) using any method of Family Planning shows a clear clustering of high values in the districts of western Uttar Pradesh, eastern and northern districts of Rajasthan, and northern and central districts of Madhya Pradesh. On the other hand, there were districts in the Bihar and north eastern Uttar Pradesh with substantially lower percentage for the same. The number of districts has shrunk for the clustering of both high and low values in NFHS 5. There is striking geographical clustering of high Percentage of women who had four or more ANC visits in majority districts of Madhya Pradesh and eastern districts of Rajasthan whereas clustering of substantially low percentage is visible in eastern districts of Bihar, Bundelkhand and Terai regions of Uttar Pradesh. There is significant improvement in Uttar Pradesh, and Madhya Pradesh in NFHS 5. On the Percentage of Births with three and more children, a robust high clustering is observed in the districts of eastern Uttar Pradesh and Eastern Bihar. On contrary, there is a significant number of districts in Madhya Pradesh and south-eastern districts of Rajasthan with lower percentage of Births with three and more children. This scenario has improved significantly in Uttar Pradesh over 2016–2021 period (**Figs 10 and 11**).

**Fig 12:**
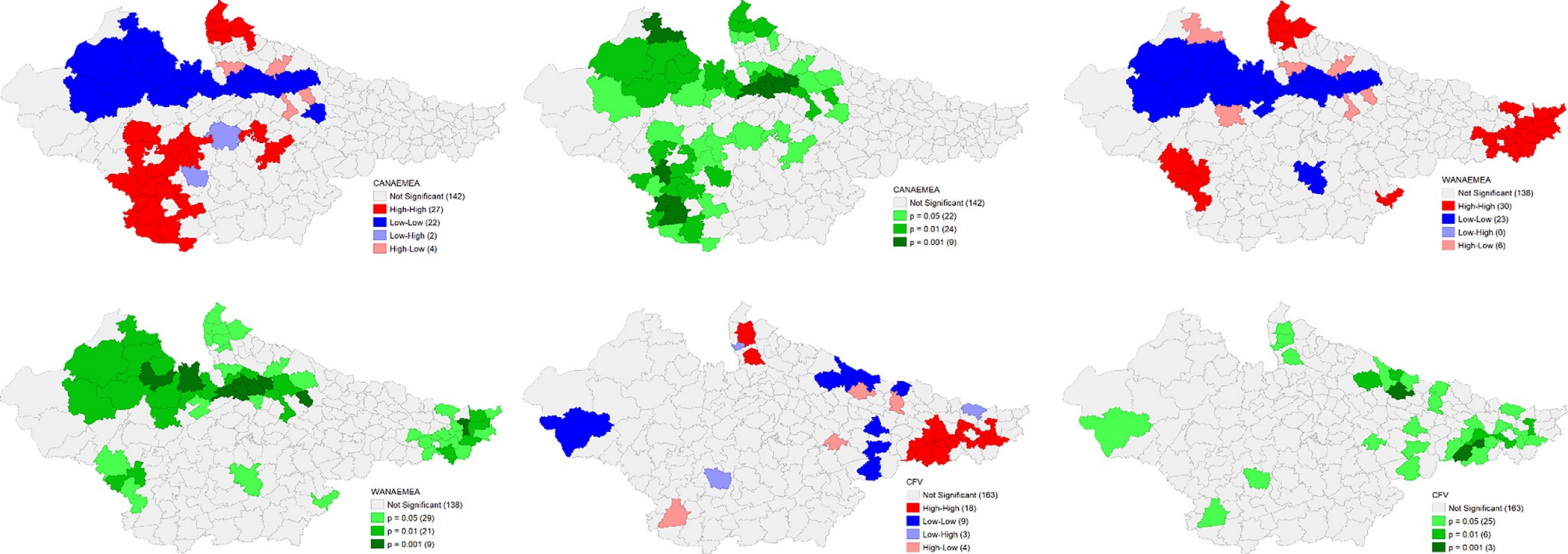
Univariate LISA (Cluster and significance) maps depicting spatial clustering and spatial outliers of District Composite Health Profile (DCHP4), based on NFHS-4. A. Univariate LISA Cluster map of Percentage of children having any anaemia (<11.0 g/d) across 197 districts in selected EAG States, 2015–16. B. Univariate LISA Significance map of Percentage of children having any anaemia (<11.0 g/d) across 197 districts in selected EAG States, 2015–16. C. Univariate LISA Cluster map of Percentage of women having any anaemia (<12.0 g/dl) across 197 districts in selected EAG States, 2015–16. D. Univariate LISA Significance map of Percentage of women having any anaemia (<12.0 g/dl) across 197 districts in selected EAG States, 2015–16. E. Univariate LISA Cluster map of Percentage of children with full vaccination across 197 districts in selected EAG States, 2015–16. F. Univariate LISA Significance map of Percentage of children with full vaccination across 197 districts in selected EAG States, 2015–16.

**Fig 13:**
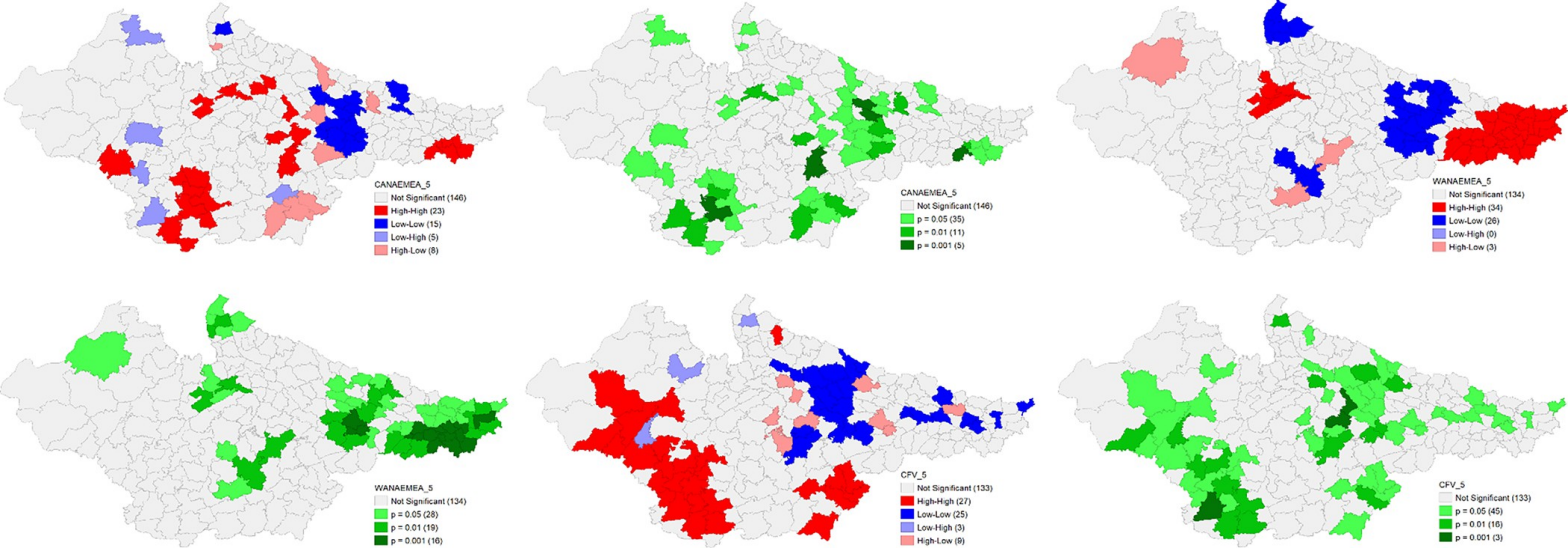
Univariate LISA (Cluster and significance) maps depicting spatial clustering and spatial outliers of District Composite Health Profile (DCHP 5), based on NFHS-5. A. Univariate LISA Cluster map of Percentage of children having any anaemia (<11.0 g/d) across 197 districts in selected EAG States, 2019–21. B. Univariate LISA Significance map of Percentage of children having any anaemia (<11.0 g/d) across 197 districts in selected EAG States, 2019–21. C. Univariate LISA Cluster map of Percentage of women having any anaemia (<12.0 g/dl) across 197 districts in selected EAG States, 2019–21. D. Univariate LISA Significance map of Percentage of women having any anaemia (<12.0 g/dl) across 197 districts in selected EAG States, 2019–21. E. Univariate LISA Cluster map of Percentage of children with full vaccination across 197 districts in selected EAG States, 2019–21. F. Univariate LISA Significance map of Percentage of children with full vaccination across 197 districts in selected EAG States, 2019–21.

The univariate LISA cluster map of Percentage of children having any anaemia (<11.0 g/d) shows a clear clustering for low values in the majority number of districts of Rajasthan and central Uttar Pradesh. A striking geographical clustering is also visible for high percentage of anaemia among children in the districts of south-eastern Madhya Pradesh and western Uttar Pradesh adjoining Uttarakhand. There is a significant improvement for high incidence of child anaemia in Madhya Pradesh and Uttar Pradesh over 2016–21 period. There are districts in eastern Bihar, north western Uttar Pradesh and south western Madhya Pradesh with substantially high percentage of women having any anaemia (<12.0 g/dl), while lower proportion in Rajasthan and central Uttar Pradesh. Significant improvement on high incidence for the same is observed in all the states with clear geographical clustering for low incidence in the districts of eastern Uttar Pradesh. Interestingly, there are many districts in south and eastern Bihar with significantly high percentage of children with full vaccination in NFHS 4. Surprisingly, in NFHS 5, high percentage of full vaccination is reported from districts of Madhya Pradesh and Southern Rajasthan and clustering of lower percentage from central Uttar Pradesh and some districts of Bihar (**Figs 12 and 13**).

**Fig 14:**
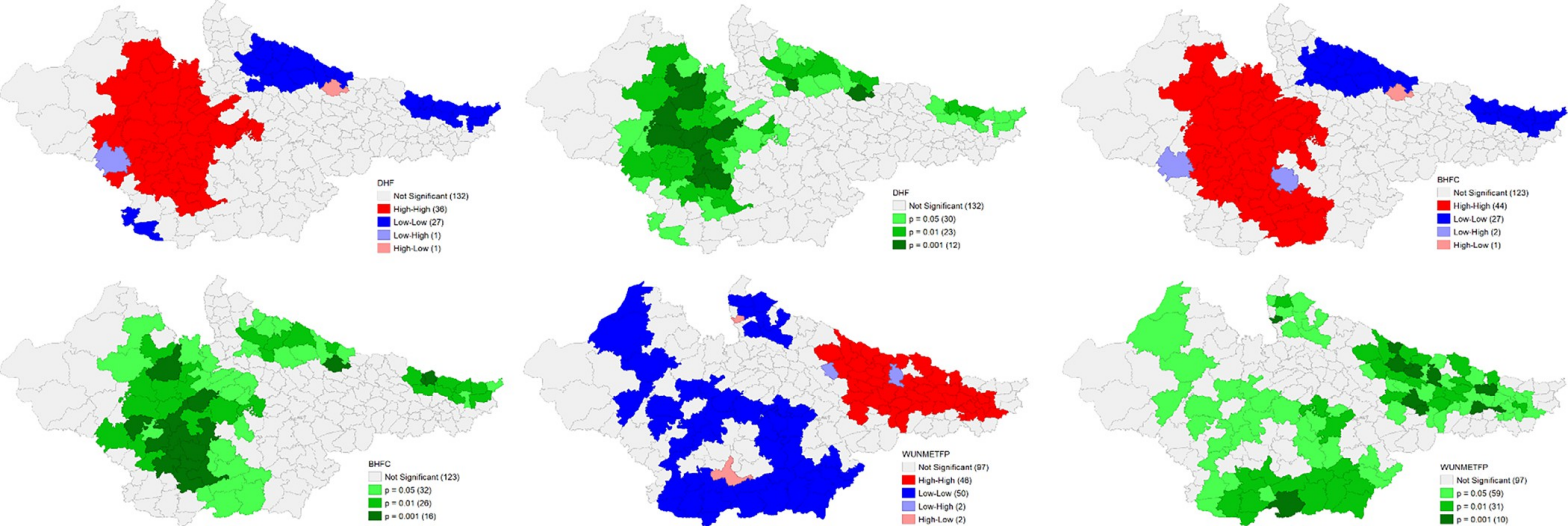
Univariate LISA (Cluster and significance) maps depicting spatial clustering and spatial outliers of District Composite Health Profile (DCHP4), based on NFHS-4. A. Univariate LISA Cluster map of Percentage of Births delivered in a health facility across 197 districts in selected EAG States, 2015–16. B. Univariate LISA Significance map of Percentage of Births delivered in a health facility across 197 districts in selected EAG States, 2015–16. C. Univariate LISA Cluster map of Percentage of Deliveries assisted by health personnel across 197 districts in selected EAG States, 2015–16. D. Univariate LISA Significance map of Percentage of Deliveries assisted by health personnel across 197 districts in selected EAG States, 2015–16. E. Univariate LISA Cluster map of Percent of currently married women (15–49) with total unmet need of Family Planning across 197 districts in selected EAG States, 2015–16. F. Univariate LISA Significance map of Percent of currently married women (15–49) with total unmet need of Family Planning across 197 districts in selected EAG States, 2015–16.

**Fig 15:**
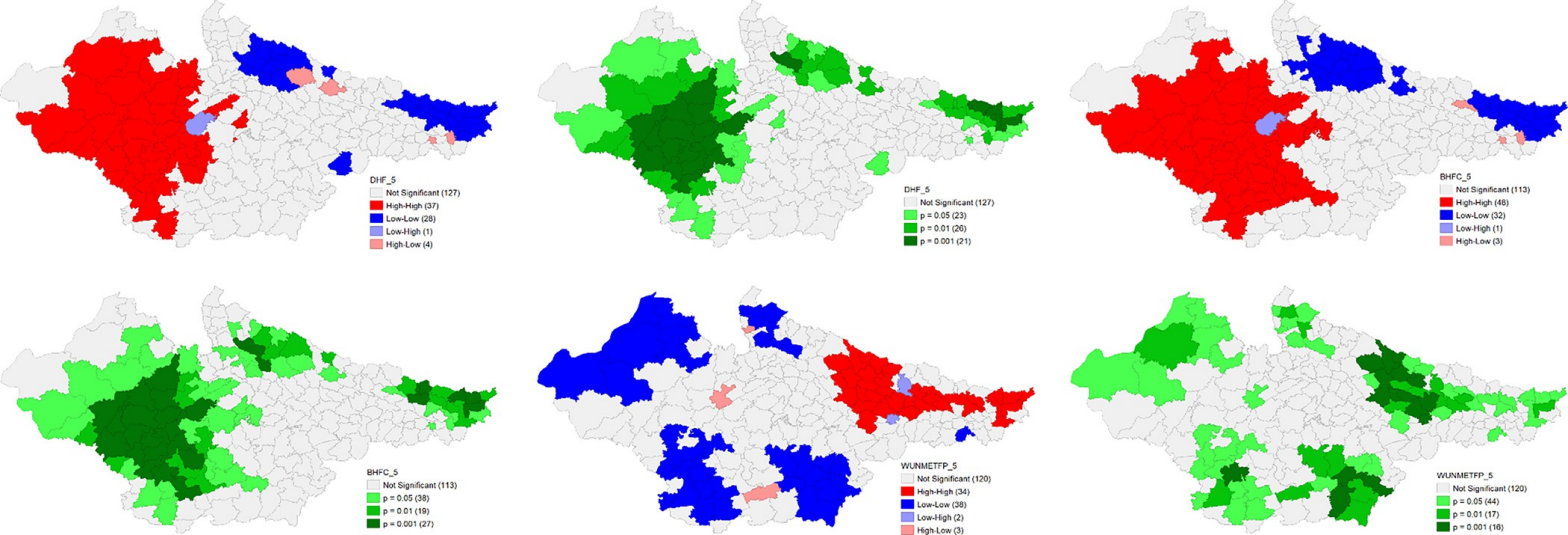
Univariate LISA (Cluster and significance) maps depicting spatial clustering and spatial outliers of District Composite Health Profile (DCHP5), based on NFHS-5. A. Univariate LISA Cluster map of Percentage of Births delivered in a health facility across 197 districts in selected EAG States, 2019–21. B. Univariate LISA Significance map of Percentage of Births delivered in a health facility across 197 districts in selected EAG States, 2019–21. C. Univariate LISA Cluster map of Percentage of Deliveries assisted by health personnel across 197 districts in selected EAG States, 2019–21. D. Univariate LISA Significance map of Percentage of Deliveries assisted by health personnel across 197 districts in selected EAG States, 2019–21. E. Univariate LISA Cluster map of DCHP5 and Percent of currently married women (15–49) with total unmet need of Family Planning across 197 districts in selected EAG States, 2019–21. F. Univariate LISA Significance map of Percent of currently married women (15–49) with total unmet need of Family Planning across 197 districts in selected EAG States, 2019–21.

The univariate LISA cluster map of Percentage of Births delivered in a health facility shows low scores in the majority number of districts in central and Rohilkhand region of Uttar Pradesh and northern Bihar bordering Nepal. On contrary, a majority of districts of Rajasthan and Madhya Pradesh were characterised by high percentage for the same. There is a little improvement in Uttar Pradesh over 2016–21 period. The spatial clustering pattern is strikingly similar for the Percentage of Deliveries assisted by health personnel. The pattern is almost identical in NFHS 5 and show almost negligible improvement. Findings also suggest interesting geographical clustering of high Percent of currently married women (15–49) with total unmet need of Family Planning in majority number of districts of Bihar and eastern Uttar Pradesh while low percentage for the same in the majority number of districts in Madhya Pradesh, western districts of Rajasthan and western Uttar Pradesh. The situation improved a little over 2016–21 period (**Figs 14 and 15**).

**Fig 16:**
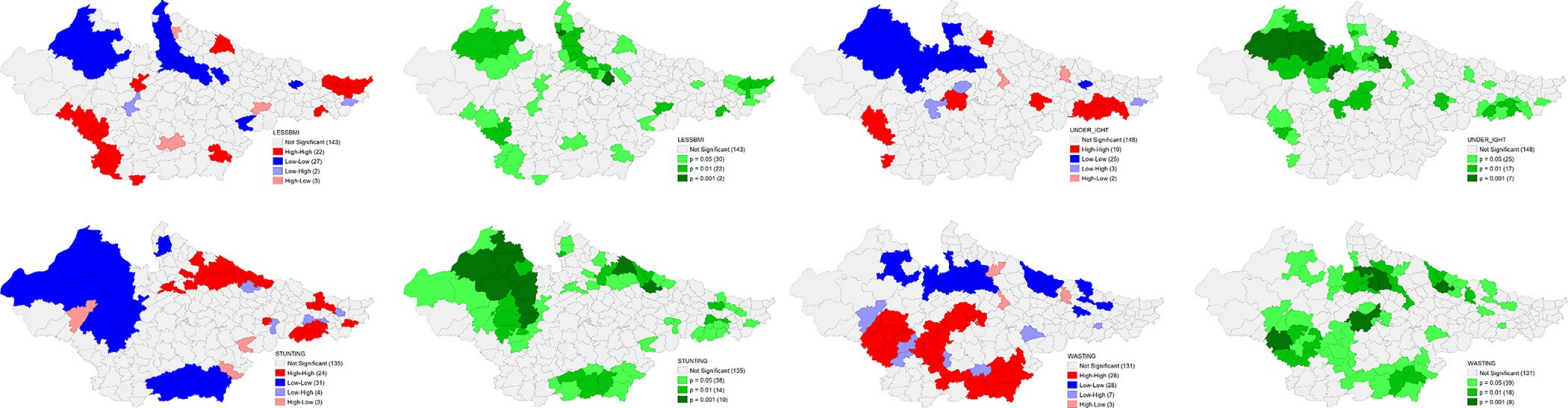
Univariate LISA (Cluster and significance) maps depicting spatial clustering and spatial outliers of District Composite Health Profile (DCHP4), based on NFHS-4. A. Univariate LISA Cluster map of Percentage of Women with BMI <18.5 (total thin) across 197 districts in selected EAG States, 2015–16. B. Univariate LISA Significance map of Percentage of Women with BMI <18.5 (total thin) across 197 districts in selected EAG States, 2015–16. C. Univariate LISA Cluster map of Underweight across 197 districts in selected EAG States, 2015–16. D. Univariate LISA Significance map of Underweight across 197 districts in selected EAG States, 2015–16. E. Univariate LISA Cluster map of Stunting across 197 districts in selected EAG States, 2015–16. F. Univariate LISA Significance map of Stunting across 197 districts in selected EAG States, 2015–16. G. Univariate LISA Cluster map of Wasting across 197 districts in selected EAG States, 2015–16. H. Univariate LISA Significance map of Wasting across 197 districts in selected EAG States, 2015–16.

**Fig 17:**
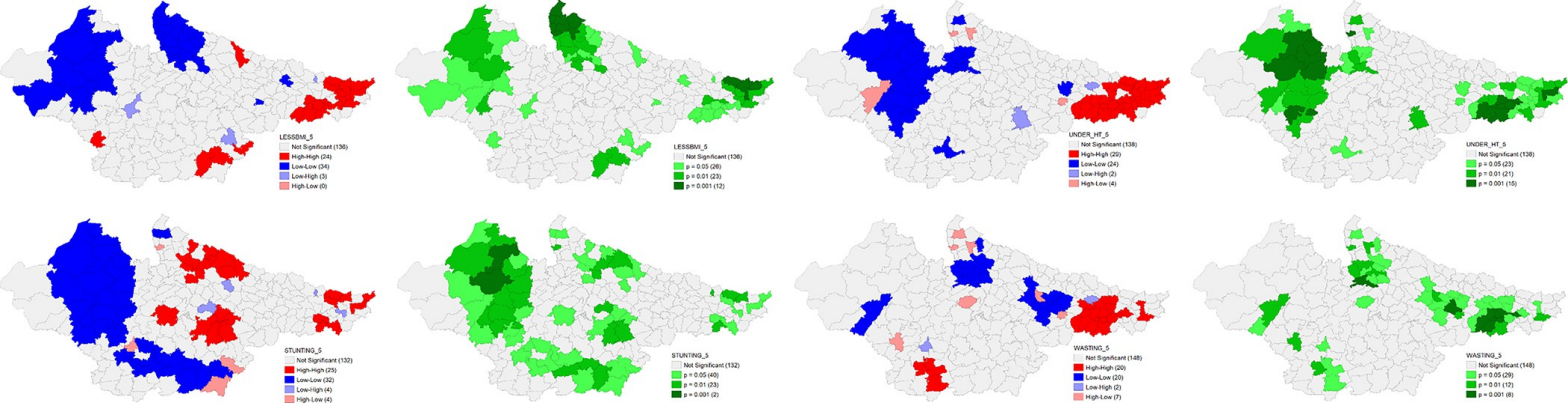
Univariate LISA (Cluster and significance) maps depicting spatial clustering and spatial outliers of District Composite Health Profile (DCHP5), based on NFHS-5. A. Univariate LISA Cluster map of Women with BMI <18.5 (total thin) across 197 districts in selected EAG States, 2019–21. B. Univariate LISA Significance map of Women with BMI <18.5 (total thin) across 197 districts in selected EAG States, 2019–21. C. Univariate LISA Cluster map of Underweight across 197 districts in selected EAG States, 2019–21. D. Univariate LISA Significance map of Underweight across 197 districts in selected EAG States, 2019–21. E. Univariate LISA Cluster map of DCHP5 and Stunting across 197 districts in selected EAG States, 2019–21. F. Univariate LISA Significance map of Stunting across 197 districts in selected EAG States, 2019–21. G. Univariate LISA Cluster map of Wasting across 197 districts in selected EAG States, 2019–21. H. Univariate LISA Significance map of Wasting across 197 districts in selected EAG States, 2019–21.

Findings also suggest that there are substantially high levels of total thin women in the eastern districts of Bihar, Rohilkhand region of Uttar Pradesh and districts of Malwa region in NFHS 4. As per NFHS 5, clustering of districts for high incidence has increased in Bihar whereas it significantly decreased in Madhya Pradesh. High incidence of underweight children was reported from districts in south Bihar, and Malwa region of Madhya Pradesh whereas low levels were reported from majority number of districts in Rajasthan. The situation has significantly improved in the districts with low incidence of underweight children in all the states but worsened in other districts of Bihar in NFHS 5. A striking geographical clustering of high incidence of stunting is visible in the central and Terai districts of Uttar Pradesh and Kaimur district of Bihar whereas for low stunting is clearly visible in the districts of Rajasthan and southern Madhya Pradesh. But there is a geographical clustering of high incidence of high stunting in the Bundelkhand adjoining districts in Madhya Pradesh. This clustering of high stunting improved marginally in the districts of central Uttar Pradesh and south Bihar. High Wasting among children is visible in the districts of south Rajasthan, central and south-eastern Madhya Pradesh while low incidence is visible in Western and Terai belt of Uttar Pradesh, and some districts of Eastern Rajasthan, and northern Bihar. There is a remarkable improvement over 2016–21 period in the majority number of districts in Madhya Pradesh. On the contrary, a striking geographical clustering in some districts of eastern Uttar Pradesh and south Bihar is also visible (**Figs 16 and 17**).

### Moran-*I* test results

The Moran-*I* statistics for DCHP and each of the indicators for both the survey rounds has been presented in the Table 3 for accounting the magnitude of geo-spatial clustering. Findings on DCHP suggest that a substantial amount of clustering is present in 2015–16 (Moran-*I* = 0.55), which further improved in 2019–21 (Moran-*I* = 0.66). Moran’s *I* for the indicators of DCHP i.e. for currently married women (15–49) with total unmet need of Family Planning, deliveries assisted by Health Personnel, and births with 3 and more children in both the survey rounds also show interesting results. The highest geo-spatial clustering was observed for the percentage of currently married women (15–49) with total unmet need of Family Planning (Moran-*I* = 0.69) during NFHS 4 while Percentage of Deliveries assisted by Health Personnel (Moran-*I* = 0.65) has the maximum degree of clustering in NFHS 5. On the contrary, insignificant amount of clustering was found for Percentage of Children with Full Vaccination in both the survey rounds. It is important to note geo-spatial clustering has improved for Percentage of Women who had four or more ANC visits, Percentage of Women with BMI <18.5 (total thin), Percentage of Births delivered in a Health Facility, Percentage of Deliveries assisted by Health Personnel, and Weight for Age for Weight (Underweight) below -2 SD.

**Table 3:** Moran-*I* statistics for the DCHP and indicators the selected EAG states of India, 2016–2021.

Variables	2015–16	2019–21
District Composite Health Profile (DCHP)	0.551	0.663
Percentage of currently married women (15–49) with total unmet need of Family Planning	0.695	0.573
Percent of currently married women (15–49) using any method of Family Planning	0.627	0.474
Percentage of Women who had four or more ANC visits	0.497	0.626
Percentage of Women having any anaemia (<12.0 g/dl)	0.518	0.503
Percentage of Women with BMI <18.5 (total thin)	0.401	0.512
Percentage of Births delivered in a Health Facility	0.505	0.571
Percentage of Deliveries assisted by Health Personnel	0.577	0.653
Percentage of Births with 3 and more Children	0.625	0.582
Percentage of Children with Full Vaccination	0.268	0.301
Percentage of Children with Height for Age (Stunting) below -2 SD	0.471	0.414
Percentage of Children with Weight for Height (Wasting) below -2 SD	0.444	0.352
Weight for Age for Weight (Underweight) below -2 SD	0.457	0.535
Percentage of Children having any Anaemia (<11.0 g/d)	0.447	0.203

Source: Calculated based on NFHS 4 (2015–16) and NFHS 5 (2019–21).

## Discussion

This study is a novel attempt to understand the progress related to reproductive health status using composite indices at the district level across the selected EAG states i.e. Uttar Pradesh, Bihar, Madhya Pradesh, and Rajasthan. The composite measure of population health status was generated by incorporating critical reproductive and child health indicators from all major demographic and health domains i.e. fertility, maternal health, and child health. Developing composite health index based on relevant indicators is a true representation of overall reproductive and child health status that play a key role in explaining vital health indicators i.e. fertility and mortality rates. In addition to this, this is also a fresh attempt in understanding the critical research questions on how spatio-temporal perspective can be helpful in understanding clustering of key health indicators as well as composite index scores using nationally representative data for two consecutive survey rounds in the high burden states. Another significance of this study is that unit of analysis is the district which is the lowest possible geographical as well as administrative level where all the health interventions are implemented by government agencies in India [41]. The EAG states under the study constitute nearly 40 percent of India’s population and around 80 percent of total EAG states’ population [45]. Trend analysis of vital health indicators shows that though major health parameters in the selected states are sluggish than the national average, nevertheless, condition has been improving over the years [30, 31, 47–49, 72, 74–76]. Maternal health status is laggard in these states if compared to national average with slow amount of improvement. Major improvement was noticed post 2010 when economic leverage started translating in to public health gains and enhancement in critical health infrastructure. The identified risk factors demonstrate the importance of improving the quality of pregnancy care and most notably, the risk conferred by poor socioeconomic status could be mitigated by universal access to healthcare at primary, secondary and tertiary levels [77]. The EAG states with relatively sluggish demographic transition have the highest burden of infant deaths in India accompanied by stark regional differences [14]. There are identifiable regions with high poverty and high infant and under-five mortality, high child malnutrition, and regions with low poverty and low infant and under-five mortality and high female literacy. However, there is consistent decline in the incidence of infant mortality in such regions over the years [10].

The key component of the study was developing composite health index i.e. DCHP for two consecutive survey rounds at district level. This study attempts to combine the key indicators of fertility, maternal health and infant/chid health in to a single score and it is an important parameter of identifying the spatial variation across the districts and translated improvement and dis-improvements. Findings suggest that though geographical clustering is highly identical to the existing political boundaries of the states under the study, nevertheless, there are stark intra-state variability in the prevalence of DCHP and its indicators.

In terms of DCHP, the districts in Bihar and Uttar Pradesh, particularly along the Terai region bordering Nepal have consistently performed poor, whereas majority districts in the north-central Rajasthan and west-central Madhya Pradesh have high scores (**S1 and S2 Tables in S1 File**). The pattern of geographical clustering largely remained same in both NFHS 4 and NFHS 5 survey rounds, albeit, number of districts in both hot spots and cold spots regions have increased in NFHS 5 with magnitude of clustering has become even stronger from NFHS 4 (**Table 3**). This clearly indicates that clustering within the high burden states present a dismal state and raise questions on health interventions and their implementation on the actual ground. Healthcare affordability, poor economic condition and associated low health spending, low literacy levels, poor health and hygiene behaviour, and social practices such as early marriages, restricted mobility of women, intra household power dynamics, role of mother in laws, health taboos, gender discrimination, etc. are some of the demand side constraints, that are usually associated with poor health outcomes [43, 78]. Healthcare affordability, particularly relating to chronic ailments erodes family income to a greater extent. Either the cost of healthcare is high or the income of the household is low, or the combination of both. Lifestyle related habits such drinking and smoking among men along with low public awareness on nutritional requirements and socially segregated food culture, particularly for children and women are the major causes of poor outcomes. Poor health condition causes loss of man-days and results in loss of livelihood due to hospitalisation. This further worsens the economic standing of families [79, 80]. Health literacy is dismally low in such districts and this manifests in low patient compliance in the early trimesters for mothers, that further aggravates to pre-natal and post-natal pregnancy complications. The poor menstrual and hygiene practices are also one of the key reasons for low performance of these indicators. Early marriages mostly in Bihar and eastern Uttar Pradesh is one of the leading causes of poor performance of these areas. In rural settings, the power dynamics within a household such as restricted mobility and freedom of expression on reproductive health is a major cause of poor outcomes in laggard districts [77, 78].

On the supply side, among various reasons for overall poor status of reproductive and child health include lack of proper healthcare like insufficient medical supplies, lack of skilled medical staffs in government hospitals, longer waiting time, particularly at primary and community hospitals, over burdening of health facilities, low patient compliance, and poor condition of transportation facilities [60, 81–83]. A chunk of rural healthcare in the low performing districts is provided by private healthcare sector and quacks. The cost of hospitalisation and diagnostic is generally high in private facilities in comparison to public funded facilities [84]. Due to low economic standing, the ANC, PNC and institutional deliveries are alarmingly low among the poor performing districts. Besides, inadequate road infrastructure restricts the access to health facilities and comes with a greater cost as people have to arrange their own vehicle, particularly for accessing the reproductive and delivery care. In terms of the key indicators of fertility, there is a strong hot and cold spots clustering in the districts of Rajasthan, and districts of Terai eastern Uttar Pradesh and Bihar, respectively. Studies suggest that within the high focus states, there is a clear association of high fertility with social confounders like early marriage age, low contraception use, and poor socio-economic profiles [71, 85, 86]. Teenage marriages are still very high in the socio-economically poor districts owning to customary practices. Such districts have also limited prevalence of contraception use due to lack of knowledge of reproductive health and family planning, and low economic status resulting in high fertility behaviour [22, 71].

On key maternal health indicators, there is a clear geographical clustering of high prevalence for good indicators in the districts of Rajasthan and Madhya Pradesh and for low prevalence in Bundelkhand region in Uttar Pradesh, Bihar and Terai districts of Uttar Pradesh. Only exception to this was percentage of women having any anaemia (<12.0 g/dl) where clear clustering in Bihar and in the districts of eastern Uttar Pradesh for high and low incidence is identifiable, respectively. There are compelling requirements to prioritise maternal education, low birthweight, low income levels and prevalent social norms and social stratification in the national and state specific policies to improve reproductive health in such states with district specific plans and policies like Janani Surakha Yojana [56, 81]. Besides, the role of ASHA, ANMs and Anganwadis as the first point of contact need to be strengthened in such pockets [87]. Nevertheless, there has been a significant reduction in inequality that can be attributed to the various pro-poor policies and cash incentive schemes successfully launched in recent years and community-level involvement with due consideration of contextual factors [20, 27, 88].

Another key component of this study was understanding the spatial clustering pattern of key child/infant health indicators. Findings suggest that on account of negative indicators, there is again clear geographical clustering of high incidence in districts of Bihar and low in the districts of central and eastern Rajasthan, western Uttar Pradesh and western Madhya Pradesh. Despite being a common cultural region, there is clear clustering in the districts of western Bihar and eastern Uttar Pradesh for high wasting and low wasting incidence, respectively. This clearly shows that the role of government as policy formulator and implementer is critical along with social norms and practices. In terms of percentage of children having any anaemia (<11.0 g/d), there is no regional or state boundary based clear clustering of districts, rather there are pockets with high prevalence within the state. Our findings on these key indicators is corroborated by many district level studies on infant and child mortality [9, 17–19, 23, 82, 89]. This highlights the role of district as a unit of health administration and important enabler along with the state administration.

The National Health Policy (2017) envisioned for reducing IMR to 28 by 2019 and under five mortalities to 23 by 2015. This policy also aims for reducing MMR to 100 by 2020 [90]. The National Population Policy (2000) aims for reducing the IMR to below 30 per 1000 live births and MMR to below 100 along with addressing the unmet need of family planning, achieving the universal immunization and 80 percent institutional deliveries and 100 percent deliveries by trained persons [91]. The Janani Suraksha Yojana (JSY) focuses on the poor pregnant woman with special dispensation. It is being implemented with the objective of reducing maternal and neo-natal mortality by promoting institutional delivery among the poor pregnant women by focusing on High Performing States (HPS) and Low Performing States (LPS) comprising Uttar Pradesh, Bihar, Madhya Pradesh, and Rajasthan, etc. [92]. Besides, government of India has taken initiatives like Janani Shishu Suraksha Karyakaram (JSSK) that ensures every pregnant woman to have cost free delivery care, including for caesarean section, in public health institutions along with the provision of free transport, diagnostics, medicines, other consumables, diet and blood. The Surakshit Matratva Ashwasan (SUMAN) aims to provide assured, dignified, respectful and quality healthcare at no cost and zero tolerance for denial of services for every woman and newborn visiting the public health facility. Other programs include the Pradhan Mantri Surakshit Matritva Abhiyan (PMSMA) for free ANC visit on 9^th^ day every month, LaQshya (Labour room Quality improvement Initiative) to improve the quality of care in Labour room and Maternity operation theatres, and Birth Microplanning and Birth Preparedness by Skilled birth attendance (SBA) trained ANMs, with other many ongoing programs [93]. Government of India adopted the Reproductive, Maternal, New-born, Child and Adolescent Health (RMNCH+A) framework in 2013 aiming to reduce causes of mortality and morbidity among women and children, along with understanding the delays in accessing and utilizing health care services [94–96]. Besides, Mission Parivar Vikas has been launched by government of India for substantially increasing access to contraceptives and family planning services in 146 high fertility districts with fertility rate above 3 in specifically in seven high focus EAG states [97].

In the light of current findings, it is imperative to expedite aforementioned health interventions in the high focus districts within these high focus states. High policy ambitions needs to be met with robust implementation on ground with a vision of universal coverage and accurate mechanism and methods of consistent monitoring [2, 7, 8, 41]. One health indicator might be highly related with another but priority of individual member might be different due to contextual confounders [98]. Thus, indexing through key health indicators provides a good picture of priority locations for universal health access that should also consider geographical space as important predictor. Health studies in the context of factors such as wealth and literacy are useful but not sufficient. The associated Individual level indicators (**S3 and S4 Tables in S1 File**) and their compounded setting need to be seen in complementarity to key maternal and child health policies and programmes. The Focus on the lowest possible spatial unit in these high focus states will help in addressing the dismal state of reproductive and child health and realising SDG-3 goal for India by 2030.

### Limitations of the study

One of the key limitations of our study was lack of district level data on the key indicators in NFHS 3, 2 and 1 rounds. Longer duration data on the said variables could have enhanced the implication of results. Another major limitation was focus of our study on the four major states within the EAG states. Our focus was only on the high burden states with significant population. The exclusion of major environmental factors such as climate, rainfall, temperature, economic activities, etc. was another limitation due to lack of data for geographical space-based analysis.

## Supporting information

S1 File Supplementary tables. (DOCX)
