# Supplementary material for: Reproductive and child health transition among selected empowered action groups states of India: A district-level analysis
Source: PLoS One. 2024 Jun 10;19(6):e0301587. doi: 10.1371/journal.pone.0301587 (PMC11164384; doi:10.1371/journal.pone.0301587)
Supplement: S1 File — (DOCX) [file pone.0301587.s001.docx]

**Supplementary Data Sets**

**Table S1**

| **Bottom 10th Percentile DISTRICTS** | **DCHP (NFHS 4) SCORES** | **Top 10th Percentile DISTRICTS** | **DCHP (NFHS 4) SCORES** |
| --- | --- | --- | --- |
| Bahraich | 0.1419 | Ganganagar | 0.8042 |
| Shrawasti | 0.1675 | Jhunjhunun | 0.7822 |
| Balrampur | 0.1680 | Jaipur | 0.7598 |
| Barwani | 0.1930 | Sikar | 0.7435 |
| Purnia | 0.2488 | Indore | 0.7283 |
| Kishanganj | 0.2600 | Gautam Buddha Nagar | 0.7246 |
| Siddharth Nagar | 0.2622 | Hanumangarh | 0.6983 |
| Sitamarhi | 0.2737 | Total | 0.6826 |
| Purba Champaran | 0.2839 | Kota | 0.6742 |
| Kheri | 0.2851 | Ghaziabad | 0.6688 |
| Darbhanga | 0.2907 | Hapur | 0.6672 |
| Udaipur | 0.2932 | Agra | 0.6620 |
| Pratapgarh | 0.2946 | Dausa | 0.6595 |
| Alirajpur | 0.3068 | Jabalpur | 0.6537 |
| Katihar | 0.3088 | Bikaner | 0.6526 |
| Kaushambi | 0.3094 | Nagaur | 0.6495 |
| Sheohar | 0.3113 | Mahamaya Nagar | 0.6389 |
| Pashchim Champar | 0.3129 | Ajmer | 0.6264 |
| Gonda | 0.3149 | Narsimhapur | 0.6212 |
|  |  | Meerut | 0.6194 |
|  |  | Raisen | 0.6173 |

**Table S2**

| **Bottom 10th Percentile DISTRICTS** | **DCHP (NFHS 5) SCORES** | **Top 10th Percentile DISTRICTS** | **DCHP (NFHS 5) SCORES** |
| --- | --- | --- | --- |
| Purnia | 0.1633 | Jaipur | 0.7560 |
| Katihar | 0.1980 | Kota | 0.7559 |
| Araria | 0.1991 | Indore | 0.7319 |
| Saharsa | 0.2129 | Nagaur | 0.7220 |
| Kishanganj | 0.2206 | Udaipur | 0.7200 |
| Sitamarhi | 0.2289 | Bhilwara | 0.7181 |
| Jehanabad | 0.2296 | Bijnor | 0.7157 |
| Bhojpur | 0.2443 | Pratapgarh | 0.7125 |
| Bahraich | 0.2451 | Jodhpur | 0.7120 |
| Balrampur | 0.2548 | Jhunjhunun | 0.7074 |
| Purba Champaran | 0.2625 | Chittaurgarh | 0.6982 |
| Patna | 0.2961 | Ganganagar | 0.6935 |
| Banda | 0.3034 | Neemuch | 0.6926 |
| Arwal | 0.3066 | Meerut | 0.6897 |
| Khagaria | 0.3067 | Guna | 0.6893 |
| Lakhisarai | 0.3075 | Narsimhapur | 0.6883 |
| Saran | 0.3116 | Barmer | 0.6826 |
| Sheohar | 0.3171 | Sikar | 0.6794 |
| Madhepura | 0.3179 | Tikamgarh | 0.6780 |
|  |  | Ghaziabad | 0.6776 |

**Table S3**

**Correlation Matrix of Indicators, NFHS 5**

| **Variables** | **1** | **2** | **3** | **4** | **5** | **6** | **7** | **8** | **9** | **10** | **11** | **12** | **13** |
| --- | --- | --- | --- | --- | --- | --- | --- | --- | --- | --- | --- | --- | --- |
| (1) Percent of currently married women (15-49) using any method of Family Planning | 1 |  |  |  |  |  |  |  |  |  |  |  |  |
| (2) Percentage of women who had four or more ANC visits | 0.581*** | 1 |  |  |  |  |  |  |  |  |  |  |  |
| (3) Percentage of Deliveries assisted by health personnel | 0.427*** | 0.603*** | 1 |  |  |  |  |  |  |  |  |  |  |
| (4) Percentage of Births delivered in a health facility | 0.444*** | 0.576*** | 0.923*** | 1 |  |  |  |  |  |  |  |  |  |
| (5) Percentage of Births with three and more children | -0.409*** | -0.658*** | -0.705*** | -0.635*** | 1 |  |  |  |  |  |  |  |  |
| (6) Percentage of children with full vaccination | 0.498*** | 0.435*** | 0.327*** | 0.358*** | -0.340*** | 1 |  |  |  |  |  |  |  |
| (7) Stunting | -0.292*** | -0.436*** | -0.496*** | -0.473*** | 0.525*** | -0.244*** | 1 |  |  |  |  |  |  |
| (8) Wasting | 0.007 | -0.163** | -0.082 | -0.076 | 0.163** | 0.02 | 0.001 | 1 |  |  |  |  |  |
| (9)Underweight | -0.207*** | -0.375*** | -0.366*** | -0.358*** | 0.426*** | -0.103 | 0.595*** | 0.657*** | 1 |  |  |  |  |
| (10) Percentage of children having any anaemia (<11.0 g/d) | 0.145** | 0.041 | -0.001 | -0.022 | -0.016 | 0.121* | 0.191*** | 0.03 | 0.045 | 1 |  |  |  |
| (11) Percentage of women having any anaemia (<12.0 g/dl) | -0.081 | -0.237*** | -0.278*** | -0.266*** | 0.265*** | 0.043 | 0.254*** | 0.178** | 0.341*** | 0.467*** | 1 |  |  |
| (12) Percentage of Women with BMI <18.5 (total thin) | -0.267*** | -0.228*** | -0.206*** | -0.264*** | 0.164** | -0.188*** | 0.326*** | 0.205*** | 0.460*** | 0.039 | 0.383*** | 1 |  |
| (13) Percent of currently married women (15-49) with total unmet need of Family Planning | -0.890*** | -0.485*** | -0.269*** | -0.307*** | 0.328*** | -0.534*** | 0.252*** | -0.059 | 0.144** | -0.188*** | -0.064 | 0.218*** | 1 |
| **** p<0.01, ** p<0.05, * p<0.1* |  |  |  |  |  |  |  |  |  |  |  |  |  |

**Table S4**

**Correlation Matrix of Indicators, NFHS 4**

| **Variables** | **1** | **2** | **3** | **4** | **5** | **6** | **7** | **8** | **9** | **10** | **11** | **12** | **13** |
| --- | --- | --- | --- | --- | --- | --- | --- | --- | --- | --- | --- | --- | --- |
| (1) Percent of currently married women (15-49) using any method of Family Planning | 1 |  |  |  |  |  |  |  |  |  |  |  |  |
| (2) Percentage of women who had four or more ANC visits | 0.638*** | 1 |  |  |  |  |  |  |  |  |  |  |  |
| (3) Percentage of Deliveries assisted by health personnel | 0.463*** | 0.541*** | 1 |  |  |  |  |  |  |  |  |  |  |
| (4) Percentage of Births delivered in a health facility | 0.440*** | 0.524*** | 0.945*** | 1 |  |  |  |  |  |  |  |  |  |
| (5) Percentage of Births with three and more children | -0.483*** | -0.592*** | -0.683*** | -0.599*** | 1 |  |  |  |  |  |  |  |  |
| (6) Percentage of children with full vaccination | 0.329*** | 0.272*** | 0.357*** | 0.436*** | -0.273*** | 1 |  |  |  |  |  |  |  |
| (7) Stunting | -0.466*** | -0.539*** | -0.504*** | -0.501*** | 0.651*** | -0.270*** | 1 |  |  |  |  |  |  |
| (8) Wasting | 0.279*** | 0.243*** | 0.374*** | 0.341*** | -0.384*** | 0.228*** | -0.289*** | 1 |  |  |  |  |  |
| (9)Underweight | -0.081 | -0.169** | -0.096 | -0.113 | 0.185*** | 0.044 | 0.471*** | 0.621*** | 1 |  |  |  |  |
| (10) Percentage of children having any anaemia (<11.0 g/d) | 0.062 | 0.249*** | 0.075 | 0.04 | -0.107 | 0.073 | 0.035 | 0.435*** | 0.474*** | 1 |  |  |  |
| (11) Percentage of women having any anaemia (<12.0 g/dl) | -0.111 | -0.006 | -0.096 | -0.048 | 0.098 | 0.181** | 0.127* | 0.422*** | 0.568*** | 0.755*** | 1 |  |  |
| (12) Percentage of Women with BMI <18.5 (total thin) | -0.211*** | -0.278*** | -0.135* | -0.137* | 0.166** | -0.012 | 0.376*** | 0.301*** | 0.594*** | 0.205*** | 0.370*** | 1 |  |
| (13) Percent of currently married women (15-49) with total unmet need of Family Planning | -0.841*** | -0.628*** | -0.441*** | -0.396*** | 0.578*** | -0.312*** | 0.526*** | -0.412*** | -0.018 | -0.235*** | -0.032 | 0.088 | 1 |
| **** p<0.01, ** p<0.05, * p<0.1* |  |  |  |  |  |  |  |  |  |  |  |  |  |
